# Heat stress in relation to sleep health among farmers: a cross-sectional study

**DOI:** 10.1186/s12889-026-26614-y

**Published:** 2026-02-13

**Authors:** Wensu Zhou, Symielle A. Gaston, Bethany T. Ogbenna, Christine G. Parks, Dale P. Sandler, Chandra L. Jackson

**Affiliations:** 1 Epidemiology Branch, National Institute of Environmental Health Sciences, Department of Health and Human Services, National Institutes of Health, 111 TW Alexander Drive, MD A3-05, NC Research Triangle Park, 27709 USA; 2 Intramural Program, National Institute on Minority Health and Health Disparities, Department of Health and Human Services, National Institutes of Health, Bethesda, MD USA

**Keywords:** Heat stress, Sleep duration, Sleep quality, Agricultural workers

## Abstract

**Background:**

Heat exposure has been linked to sleep disturbances in the general population, but farmers, who are particularly vulnerable due to outdoor occupational exposures—have been rarely studied in this context. this study aimed to investigate associations between heat stress and sleep health among farmers.

**Methods:**

We conducted cross-sectional analyses of 8,203 male farmers from Iowa (78%) and North Carolina (NC, 22%) in the Agricultural Health Study (2013–2015). Daily wet bulb globe temperatures (WBGT) from May 2013–September 2015 was used. We calculated absolute heat stress by averaging WBGT over 2/5/7 days before the interview. Relative heat stress (i.e., the difference between absolute heat stress and the 92.5th percentile of WBGT) was also calculated. WBGT was categorized by heat stress risk (low, moderate, high). Sleep outcomes included short sleep (< 7 h), daytime sleepiness (≥ 3 days/week), napping (yes), and long naps (≥ 30 min). Poisson regression with robust variance estimated adjusted prevalence ratios (PRs) and 95% confidence intervals (CIs), stratified by state.

**Results:**

Farmers averaged 63 years old (SD = 10.1); 37.8% reported short sleep, 8.1% daytime sleepiness, 44.6% napping, and 17.1% long naps. Mean absolute WBGT were 70.4 °F (SD = 6.36) in Iowa and 77.7 °F (SD = 7.83) in NC. In Iowa, moderate heat stress (2-day average) was associated with higher short sleep prevalence (PR = 1.04 [1.00–1.07]). In NC, higher absolute (2-/5-/7-day average) and relative WBGT (2-day average), as well as moderate (2-/7-day) and high (2-day) heat stress were associated with daytime napping (e.g., PR _2−day absolute WBGT_= 1.02 [1.01–1.04]). In both states, high heat stress was linked to lower prevalence of long naps (e.g., PR_Iowa, 2−day heat stress_= 0.86 [0.83–0.89]).

**Conclusions:**

Heat stress was associated with a small/weak but potentially meaningful relationship with poor self-reported sleep among farmers. Future studies using objective sleep measures are needed. Although our findings highlight the potential importance of incorporating heat mitigation and sleep health strategies into occupational safety guidelines for agricultural workers, the cross-sectional design of this study precludes causal conclusions.

**Supplementary Information:**

The online version contains supplementary material available at 10.1186/s12889-026-26614-y.

## Introduction

Sleep disturbances are prevalent in the general US population. Approximately 23.1% of adults report short sleep (< 7 h), 29.8% report trouble sleeping, and 27.2% experience daytime sleepiness [1]. Trouble sleeping is more prevalent among older adults [1], a concern given that the US population aged ≥ 65 is projected to grow by 47% by 2050 [2]. Sleep disturbances are linked to various chronic diseases and mental health conditions [3], highlighting the need to identify factors that impair sleep.

Prior studies have examined the relationship between ambient temperature and sleep health using absolute temperature measures (e.g., daily temperature values) as well as relative metrics that consider both temperature intensity and duration, which may better capture sleep-related risk by accounting for local temperature adaptation [4–6]. For instance, studies using mean or maximum daytime ambient temperature values have found associations between heat exposure and both reduced sleep duration and poorer sleep quality among samples of the general population [5, 7, 8]. One study among older adults reported that multi-day heat exposure above the 92.5th percentile was linked to shorter sleep duration [6], but research on relative daytime temperature and different aspects of sleep remains limited.

Ambient temperature alone may underestimate actual heat burden. For instance, high humidity hinders heat dissipation via sweating, increasing heat stress [9]. Heat stress involves physical strain caused by hot environments and is marked by elevated core temperature and heart rate [10]. The Heat Index (HI), combining temperature and humidity, is one measure of heat stress [11]. But it omits factors like wind speed and solar radiation, which also influence the body’s heat balance and can further modify heat stress levels. Thus, assessing heat exposure in relation to sleep health should involve indicators that more accurately reflect heat stress. The Wet Bulb Globe Temperature (WBGT) [12] - which incorporates wind speed, cloud cover, humidity, and sun angle—offers a more comprehensive measure of heat stress and is better suited for evaluating thermal conditions during hot-weather activities than ambient temperature alone. While several studies in the general adult population have used WBGT to examine heat and sleep, they have primarily focused on sleep duration [13, 14] and often relied on small sample sizes [13–15]. These studies have also reported mixed findings. For example, Matzke et al. found no significant association between nighttime WBGT and sleep duration [14], whereas another survey reported a significant association between higher WBGT and poor sleep quality [15]. The study findings likely differ due to differences in study population as prior research has rarely focused on outdoor workers, who may experience substantially higher levels of heat exposure. Studies have also differed in study design, geographic region, study time periods, and dimensions of sleep assessed, which is important since some dimensions may be more or less affected by heat exposure [13–15]. 

Farmers, particularly during peak agricultural seasons, are highly susceptible to heat-related health issues due to prolonged exposure to sun and physical exertion [16]. During farming production periods, which often overlap with the hottest months, farmers are more likely to experience elevated body core temperature and dehydration due to prolonged exposure to solar radiation [17]. Along with the increasing frequency and intensity of extreme heat events, agricultural productivity is expected to face greater threats and increased risks of heat stress and heat-related adverse health outcomes [17]. Elevated core body temperature can contribute to discomfort, and heat exposure may impair thermoregulation by skin vasodilation as well as contribute to increased wakefulness, thereby impacting sleep [18]. Combined with labor fatigue, this may heighten risk for sleep disturbances and related outcomes such as injury. However, evidence on the link between heat stress and sleep among farmers is limited. Age may modify this relationship. Since older people are more vulnerable to heat primarily due to age-related diminished thermoregulatory function and the presence of comorbidities [19]. One study showed that as daily mean temperatures rose, sleep duration declined - especially among older adults (aged over 65 years) compared to younger age groups (19 to 65 years) [20]. Prior studies examining heat stress and sleep have primarily included general adult populations and have not conducted age-stratified analyses [13–15]. Consequently, little is known about age-related differences in the heat stress–sleep relationship, particularly among farmers.

We aimed to investigate whether absolute and relative WBGT are associated with sleep duration and disturbances among US farmers and whether age modifies these associations. We hypothesized that heat stress is associated with short sleep duration, daytime sleepiness, daytime napping, and long napping duration. Further, we hypothesized that these associations are stronger among older than younger farmers.

## Methods

### Data source and study population

We used data (Releases: P1REL202210.00) from the Agricultural Health Study (AHS) [21], a prospective cohort study of more than 89,000 farmers and their spouses from Iowa and North Carolina (NC), enrolled in 1993–1997. Participants completed questionnaires at enrollment and follow-up surveys in 1999–2003, 2005–2010, 2013–2015, and 2019–2021. Participants provided implied consent at enrollment and active consent during follow-up surveys, following protocols approved by the relevant Institutional Review Boards.

This analysis used cross-sectional data from the 2013–2015 survey, which included available sleep data. Of 42,331 respondents, we excluded spouses (*n* = 18,186) and female farmers (*n* = 661) due to potential differences in heat exposure between male farmers and their spouses, and the small sample size of female farmers, which resulted in limited power to conduct sex-specific analyses. We further excluded those not living on a farm or not farming in the past year (*n* = 10,669), interviewed outside the warm season (May-September; *n* = 4,040) [22], missing key covariates or sleep data (*n* = 526), or missing WBGT data (*n* = 46). The final analytic sample included 8,203 male farmers (Iowa: *n* = 6,403; NC: *n* = 1,800). Excluded participants were generally older, but distributions of education and marital status were similar (Table S1; flowchart in Figure S1).

### Exposure assessment: heat stress

Iowa is located in the central region of the US and has a four-season climate. The average temperature during the warm season months was 67.8 °F (19.9 °C) in 2013, 66.4 °F (19.1 °C) in 2014, and 67.7 °F (19.8 °C) in 2015 [23]. The weather in NC, located along the Atlantic coast, is humid with very warm summers and moderately cold winters. During the warm season months from 2013 to 2015, average temperatures in NC ranged from 72.0 °F (22.2 °C) in 2013 to 73.0 °F (22.8 °C) in 2014, and 74.4 °F (23.6 °C) in 2015 [23]. 

Daily relative humidity and temperature data linked to the AHS were derived from satellite-based climate data provided by the North American Regional Reanalysis (NARR), a product of the National Centers for Environmental Prediction (NCEP) and the National Oceanic and Atmospheric Administration (NOAA). These data are publicly accessible and available on a daily basis (https://www.ncei.noaa.gov/products/weather-climate-models/north-american-regional). NARR data are provided at a 32-kilometer resolution, and geocoded baseline participant home addresses were matched to the nearest grid cell. Thus, daily relative humidity and temperature values were assigned at the grid-cell level and linked to each participant’s residential address, and they were used to calculate daily HIs. Next, the HI was used to calculate WBGT using: -0.0034 HI^2^ + 0.96 HI – 34 [24]. Since short-term temperature fluctuations can influence sleep [25], the critical exposure window periods were defined by the following number of days prior to the interview date: 2-day, 5-day, and 7-day moving averages. For example, a 2-day moving average was defined as the two-day average levels of WBGT exposure prior to interview day.

We calculated relative exposure to WBGT. This approach defines exposure using several percentile-based thresholds of the WBGT distribution over a specified period, and these thresholds are based on long-term, location-specific temperature records, allowing for a more accurate estimation of heat-related health impacts [26]. In a previous survey focused on daily temperature, commonly used thresholds included the 90th, 92.5th, 95th, and 97.5th percentiles [27]. Thus, we calculated the 90th, 92.5th and 95th percentiles for the WBGT series in the warm season (May to September) of 2013–2015 for each participant based on their locations. Our main analysis used the 92.5th percentile, which reflects mild to moderate heat intensity with known health effects [28]. Relative exposure was then defined as the difference between absolute WBGT and the corresponding percentile threshold.

We also categorized WBGT based on heat safety guidelines adapted for outdoor activity, which account for regional climate differences [22]. The thresholds were previously determined by U.S. region (*n* = 3) based on geographic distribution, with variations reflecting local climate adaptation [22]. This use of these thresholds has been endorsed in the Expert Consensus Statement on Exertional Heat Illness: Recognition, Management, and Return to Activity [29]. Based on the guidelines, the three heat stress exposure categories were: ‘normal activity’ (noted as ‘low risk’ in the present study; WBGT < 78.8 °F for Iowa and < 82.1 °F for NC), ‘plan intense or prolonged activity with discretion’ (moderate risk; WBGT 78.8–83.7 °F for Iowa and 82.1–86.0 °F for NC), and ‘limit or cancel outdoor activity’ (high risk; WBGT > 83.7 °F for Iowa and > 86.0 °F for NC).

### Outcome assessment: sleep duration, daytime sleepiness, and napping

Nightly sleep duration was determined based on responses to “How many hours of sleep do you get each night?”. We dichotomized responses as < 7 h vs. ≥7 h, consistent with the joint recommendations of the American Academy of Sleep Medicine and the Sleep Research Society [30]. We defined daytime sleepiness based on responses to the question “How often do you feel sleepy most of the day?”, categorized as ≥ 3 days/week vs. <3 days/week. Daytime sleepiness was used as a proxy for poor sleep quality, as its symptoms—such as untimely and unwanted sleep episodes—are indicative of poor sleep quality [31]. Two other measures, daytime napping (“Do you nap during the day?”, yes vs. no) and duration of napping among participants who reported napping (“How long do you nap?”, ≥ 30 min vs. <30 min) were also included.

### Potential effect modifier

Participants’ ages were categorized into two groups: <60 years and ≥ 60 years.

### Potential confounders

Based on a directed acyclic graph (DAG) (Figure S2), current age and marital status (both collected in the 2013–2015 survey), race and ethnicity, and educational attainment (collected at enrollment) were considered potential confounders in the analysis.

### Statistical analysis

All analyses were conducted separately for Iowa and NC due to geographic and climate differences. We used descriptive statistics (means and standard deviations [SDs] for continuous variables and counts and percentages for categories variables) to summarize sample characteristics and compared variables between states using t-tests or chi-square tests.

We estimated the relationship between heat stress (i.e., absolute WBGT, relative WBGT, and heat stress categories) and measures of sleep health, by state. Using Poisson regression with robust variance estimation, we calculated prevalence ratios (PR) and 95% confidence intervals (CIs). For absolute WBGT and relative WBGT, we assessed associations with a 1 SD increase. For categorical WBGT, the low-risk category (i.e., ‘normal activity’) served as the reference. All models were adjusted for age, race and ethnicity, marital status, and educational attainment. Effect modification by age was examined using stratified models and cross-product interaction terms.

We conducted several sensitivity analyses: (1) we applied generalized additive models (gam), fitting natural spline terms with 3 degrees of freedom, as commonly used in temperature-related health studies [32], to assess potential non-linear associations and evaluate their impact on the main findings; (2) we included female farmers with complete demographic and WBGT data in the analysis (*n* = 96 for Iowa and *n* = 54 for NC); (3) we modified the short sleep duration cut point to < 8 h versus ≥ 8 h since sleep duration is often misreported/overestimated [33] and we re-classified napping duration using ≥ 1 h as the threshold [34]; and (4) we modified the thresholds for relative heat stress measurements by using the 90th and 95th percentiles of WBGT. This was employed to assess whether using lower or higher thresholds than those in the main analysis would alter the associations between WBGT and measures of sleep health. We also sought to determine whether the association between heat stress and sleep were further influenced by different levels of relative heat stress: (5) we adjusted for daytime napping in sleep duration models and for sleep duration in napping models and (6) we used mean warm-season WBGT in the survey year as the main exposure to assess whether the short-term exposure metrics used in the primary analysis yielded robust results. This approach was motivated by the fact that sleep measures obtained from the questionnaire did not reflect a specific time window.

## Results

### Study population characteristics

Among the 8,203 farmers, 78.1% (*n* = 6,403) were from Iowa and 21.9% (*n* = 1,800) from NC, with a mean age of 62.5 years (SD = 9.84) in Iowa and 64.8 years (SD = 10.6) in NC (Table 1). Most participants were White (99.1%), with a higher proportion in Iowa (99.8%) than NC (96.7%). Age, marital status, and education significantly differed by state (*p* < 0.05).

**Table 1:** Sociodemographic characteristics, sleep health, and heat stress measures among male farmers, 2013–2015

	Total *N* = 8,203 (100.0%)	Iowa *n* = 6,403 (78.1%)	NC *n* = 1,800 (21.9%)	*P* for group comparison
Sociodemographic characteristics				
Age (years), mean (SD) ^a^	63.0 (10.1)	62.5 (9.84)	64.8 (10.6)	< 0.001^j^
Age categories				
< 50 years	651 (7.9)	520 (8.1)	131 (7.3)	< 0.001^k^
51–60 years	2612 (31.8)	2168 (33.9)	444 (24.7)	
61–70 years	2762 (33.7)	2137 (33.4)	625 (34.7)	
> 70 years	2178 (26.6)	1578 (24.6)	600 (33.3)	
Race and ethnicity ^b, c^				
Non-Hispanic White	8132 (99.1)	6392 (99.8)	1740 (96.7)	< 0.001^k^
Other	71 (0.9)	11 (0.2)	60 (3.3)	
Educational attainment ^c^				
1–8 years	147 (1.8)	103 (1.6)	44 (2.4)	< 0.001^k^
Some high school	198 (2.4)	92 (1.4)	106 (5.9)	
High school graduate or higher	7858 (95.8)	6208 (97.0)	1650 (91.7)	
Marital status ^d^				
Married/Cohabitating	7174 (87.5)	5667 (88.5)	1507 (83.7)	< 0.001^k^
Other	1029 (12.5)	736 (11.5)	293 (16.3)	
Heat stress measures				
Absolute WBGT, mean (SD)				
2-day average	72.0 (7.37)	70.4 (6.36)	77.7 (7.83)	< 0.001^j^
5-day average	71.8 (6.76)	70.1 (5.48)	77.8 (7.37)	< 0.001^j^
7-day average	71.6 (6.55)	69.9 (5.18)	77.9 (7.08)	< 0.001 ^j^
Relative WBGT, mean (SD)				
2-day average	-8.25 (6.20)	-8.25 (6.25)	-8.22 (6.01)	0.835 ^j^
5-day average	-8.46 (5.34)	-8.55 (5.36)	-8.13 (5.26)	0.003 ^j^
7-day average	-8.60 (5.00)	-8.74 (5.05)	-8.09 (4.76)	< 0.001^j^
Heat stress categories based on absolute exposure at 2-day average ^e^				
Low risk	7056 (86.0)	5874 (91.7)	1182 (65.7)	< 0.001 ^k^
Moderate risk	887 (10.8)	508 (7.9)	379 (21.1)	
High risk	260 (3.2)	21 (0.3)	239 (13.3)	
Heat stress categories based on absolute exposure at 5-day average ^e^				
Low risk	7382 (90.0)	6203 (96.9)	1179 (65.5)	< 0.001^k^
Moderate risk	634 (7.7)	186 (2.9)	448 (24.9)	
High risk	187 (2.3)	14 (0.2)	173 (9.6)	
Heat stress categories based on absolute exposure at 7-day average ^e^				
Low risk	7451 (90.8)	6292 (98.3)	1159 (64.4)	< 0.001^k^
Moderate risk	612 (7.5)	107 (1.7)	505 (28.1)	
High risk	140 (1.7)	4 (0.1)	136 (7.6)	
Sleep health measures				
Sleep duration ^f^				
≥ 7 h	5104 (62.2)	4059 (63.4)	1045 (58.1)	< 0.001^k^
< 7 h	3099 (37.8)	2344 (36.6)	755 (41.9)	
Daytime sleepiness ^g^				
< 3 days/week	7539 (91.9)	5923 (92.5)	1616 (89.8)	< 0.001^k^
≥ 3 days/week	664 (8.1)	480 (7.5)	184 (10.2)	
Daytime napping ^h^				
No	4543 (55.4)	3478 (54.3)	1065 (59.2)	< 0.001^k^
Yes	3660 (44.6)	2925 (45.7)	735 (40.8)	
Napping duration ^i^				
≥ 30 min	1401 (17.1)	1040 (16.2)	361 (20.1)	< 0.001^k^
< 30 min	6802 (82.9)	5363 (83.8)	1439 (79.9)	

Data are presented as count (percentage) or mean (standard deviation)

*NC *North Carolina

*WBGT * Wet Bulb Globe Temperature

*F *Fahrenheit

^a^ Age was measured in 2013-2015

^b^ Other racial and ethnic groups include participants who identified as Non-Hispanic Black, American Indian or Alaska Native, Asian or Pacific Islander, Hispanic/Latino of any race, multiracial, or as ‘other’ race and measured in 1993-1997

^c^ Educational attainment, race, and ethnicity were reported in 1993-1997

^d^ Other marital status categories included single, divorced or separated, and widowed (measured in 2013-2015)

^e^ Low risk indicates that normal activity is recommended: WBGT < 78.8 °F for Iowa and < 82.1 °F for NC; moderate risk indicates that planning intense or prolonged activity with discretion is recommended: WBGT 78.8–83.7 °F for Iowa and 82.1–86.0 °F for NC, and high risk indicates that limited or cancelling outdoor activity is recommended: WBGT > 83.7 °F for Iowa and > 86.0 °F for NC

^f^ Sleep duration was assessed by asking participants, “How many hours of sleep do you get each night?”. Participants could respond with: ‘less than 6 hours’, ‘6 hours to 6 hours and 59 minutes’, ’7 h to 7 hours and 59 minutes’, ‘8 hours to 8 hours and 59 minutes’, and ‘9 hours or more’

^g^ Daytime sleepiness was assessed by asking participants: “How often do you feel sleepy most of the day?”. Participants could respond with: ‘never’, ‘less than one day per month’,

‘1 to 3 days per month’, 1 to 2 days per week’, ‘3 to 5 days per week’, ‘6 to 7 days per week’

^h^ Daytime napping was assessed by asking participants, “Do you nap during the day?”. Participants could respond with: ‘yes’, and ‘no’

^i^ Napping duration was assessed by asking participants, “How long do you nap?”. Participants who reported taking naps were also asked to indicate their napping duration, response was presented as ‘less than 30 min’, and ‘ more than 30 min’

^j^ P-values obtained using t-tests

^k^ P-values obtained using Chi-square tests

WBGT indices differed between Iowa and NC. For example, using the 2-day average exposure, the mean absolute WBGTs were 70.4 °F (SD = 6.36) in Iowa and 77.7 °F (SD = 7.83) in NC. The relative WBGT were − 8.25 °F (SD = 6.25) in Iowa and − 8.22 °F (SD = 6.01) in NC. Iowa experienced more ‘normal activity’ or low risk of heat stress days than in NC (e.g., 91.7% vs. 65.7%). Short sleep duration was reported by 36.6% in Iowa and 41.9% in NC. Daytime sleepiness affected 7.5% in Iowa and 10.2% in NC. Daytime napping was reported by 45.7% in Iowa and 40.8% in NC; napping ≥ 30 min was more common in NC (20.1%) than Iowa (16.2%).

### Associations between heat stress and sleep outcomes

**Fig. 1:**
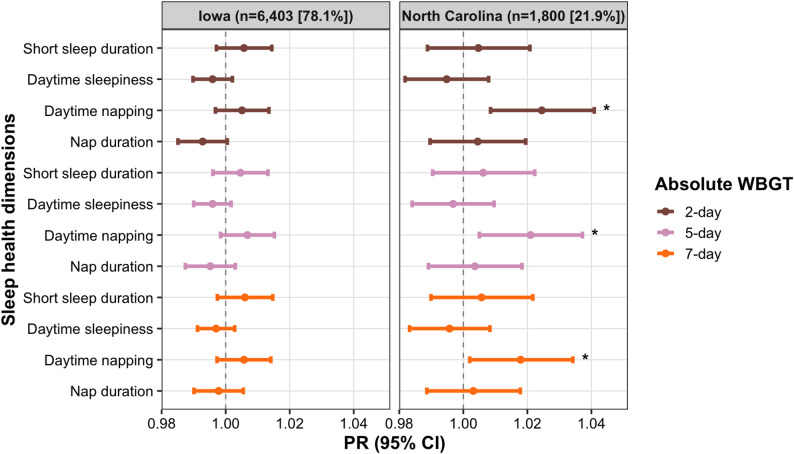
The association between each 1 SD increase in absolute WBGT and sleep health among male farmers, PR (95%CI). All models were adjusted for age, marital status, educational attainment, and race and ethnicity

**Fig. 2:**
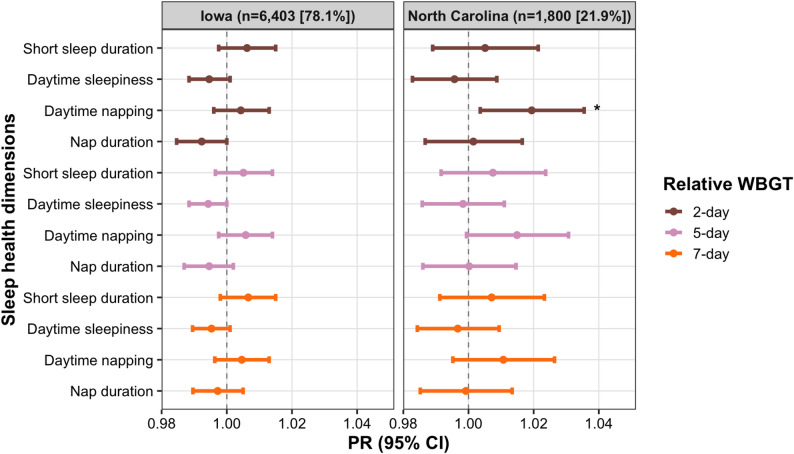
The association between each 1 SD increase in relative WBGT and sleep health among male farmers, PR (95%CI). All models were adjusted for age, marital status, educational attainment, and race and ethnicity

As shown in Figs. 1 and 2, and Table S2, absolute WBGT (2-, 5-, and 7-day averages) was not significantly associated with short sleep duration or daytime sleepiness in either state. In Iowa, WBGT was also unrelated to napping. In NC, absolute WBGT was associated with a higher likelihood of daytime napping for 2-day average exposure (PR_NC_ = 1.02 [95% CI: 1.01–1.04]), for 5-day average exposure (PR_NC_ = 1.02 [95% CI: 1.01–1.04]), and for 7-day average exposure (PR_NC_ = 1.02 [95% CI: 1.00–1.03]). A consistent finding was observed when using relative 2-day WBGT (PR_NC_ = 1.02 [95% CI: 1.00–1.04]) in NC. WBGT was not associated with napping duration in NC.

According to Table 2, in Iowa, moderate risk for heat stress, measured by the 2-day WBGT average, was associated with a higher prevalence of short sleep duration (PR = 1.04 [95% CI: 1.00–1.07]). Second, moderate and high risk for heat stress, as assessed by the 2-day average WBGT, was associated with a higher likelihood of daytime napping (e.g., PR_NC_ = 1.06 [95% CI: 1.02–1.11] for high risk) in NC. Moderate risk for heat stress, as assessed by the 7-day average WBGT, was also associated with a higher likelihood of daytime napping (PR_NC_ = 1.04 [95% CI: 1.00–1.07] for moderate risk). Third, in both NC and Iowa, high risk for heat stress across multiple WBGT exposure periods was associated with a lower prevalence of a napping duration of ≥ 30 min (e.g., PR_Iowa, 2−day WBGT_ = 0.86 [95% CI: 0.83–0.89] and PR_NC, 5−day WBGT_ = 0.95 [95% CI: 0.91–1.00]).

**Table 2:** The association between categories of WBGT ^a^ and sleep health among male farmers, PR (95%CI)

	PR (95%CI)					
	Iowa (*n* = 6,403)			NC (*n* = 1,800)		
	2-day WBGT	5-day WBGT	7-day WBGT	2-day WBGT	5-day WBGT	7-day WBGT
Sleep duration						
Moderate risk	**1.04 (1.00-1.07)**	1.03 (0.98–1.08)	1.01 (0.94–1.08)	1.01 (0.97–1.05)	1.00 (0.96–1.04)	1.02 (0.98–1.06)
High risk	1.06 (0.91–1.22)	1.04 (0.87–1.24)	0.91 (0.65–1.27)	1.01 (0.96–1.05)	1.04 (0.99–1.10)	1.04 (0.98–1.10)
Daytime sleepiness						
Moderate risk	1.01 (0.99–1.03)	0.99 (0.96–1.02)	0.98 (0.94–1.02)	0.99 (0.96–1.02)	0.99 (0.96–1.02)	0.99 (0.96–1.02)
High risk	0.97 (0.89–1.06)	1.00 (0.88–1.13)	1.16 (0.82–1.64)	0.99 (0.95–1.03)	1.01 (0.97–1.06)	0.98 (0.94–1.03)
Daytime napping						
Moderate risk	1.01 (0.98–1.04)	1.02 (0.97–1.07)	1.00 (0.94–1.07)	**1.04 (1.00-1.08)**	1.03 (0.99–1.07)	**1.04 (1.00-1.07)**
High risk	0.95 (0.83–1.10)	1.03 (0.86–1.23)	1.26 (0.95–1.67)	**1.06 (1.02–1.11)**	1.02 (0.96–1.07)	1.01 (0.95–1.07)
Napping duration						
Moderate risk	1.00 (0.97–1.02)	1.00 (0.96–1.05)	1.02 (0.96–1.08)	1.01 (0.97–1.05)	1.01 (0.97–1.05)	1.02 (0.98–1.05)
High risk	**0.86 (0.83–0.89)**	**0.89 (0.86–0.92)**	**0.89 (0.86–0.93)**	1.02 (0.98–1.07)	**0.95 (0.91-1.00)**	0.96 (0.91–1.01)

All models were adjusted for age, marital status, educational attainment, and race and ethnicity

Boldface indicates statistical significance at a two-sided p-value of 0.05

*SD *standard definition*, NC *North Carolina, *CI *confidence interval, *PR *prevalence ratio, *WBGT *Wet Bulb Globe Temperature

^a^ Low risk indicates that normal activity is recommended: WBGT < 78.8 °F for Iowa and < 82.1 °F for NC; moderate risk indicates that planning intense or prolonged activity with discretion is recommended: WBGT 78.8–83.7 °F for Iowa and 82.1–86.0 °F for NC, and high risk indicates that limited or cancelling outdoor activity is recommended: WBGT > 83.7 °F for Iowa and > 86.0 °F for NC

No significant interactions with age were detected in either state (Tables 3 and 4; P-values of test for interaction between age and WBGT indicators > 0.05).

**Table 3:** The association between each 1 SD increase in WBGT and sleep health across age groups in Iowa, PR (95%CI)

	PR (95%CI)					
	Age <60 years (n=2,688)			Age ≥60 years (n=3,715)		
	2-day WBGT	5-day WBGT	7-day WBGT	2-day WBGT	5-day WBGT	7-day WBGT
Sleep duration						
Absolute	1.00 (0.99-1.02)	1.00 (0.99-1.02)	1.00 (0.99-1.01)	1.01 (1.00-1.02)	1.01 (1.00-1.02)	1.01 (1.00-1.02)
Relative	1.00 (0.99-1.02)	1.00 (0.99-1.01)	1.00 (0.99-1.01)	1.01 (1.00-1.02)	1.01 (1.00-1.02)	1.01 (1.00-1.02)
Daytime sleepiness						
Absolute	0.99 (0.98-1.00)	0.99 (0.98-1.00)	0.99 (0.98-1.00)	1.00 (0.99-1.01)	1.00 (0.99-1.01)	1.00 (0.99-1.01)
Relative	0.99 (0.98-1.00)	0.99 (0.99-1.00)	1.00 (0.99-1.00)	1.00 (0.99-1.00)	0.99 (0.99-1.00)	1.00 (0.99-1.00)
Daytime napping						
Absolute	1.00 (0.99-1.01)	1.00 (0.99-1.01)	1.00 (0.99-1.01)	1.01 (1.00-1.02)	1.01 (1.00-1.02)	1.01 (1.00-1.02)
Relative	1.00 (0.99-1.01)	1.00 (0.99-1.01)	1.00 (0.99-1.01)	1.01 (1.00-1.02)	1.01 (1.00-1.02)	1.01 (1.00-1.02)
Napping duration						
Absolute	0.99 (0.98-1.01)	1.00 (0.99-1.01)	1.00 (0.99-1.01)	0.99 (0.98-1.00)	1.00 (0.98-1.01)	1.00 (0.99-1.01)
Relative	1.00 (0.99-1.01)	1.00 (0.99-1.01)	1.00 (0.99-1.01)	0.99 (0.98-1.00)	0.99 (0.98-1.00)	1.00 (0.98-1.01)

All models were adjusted for age, marital status, educational attainment, and race and ethnicity

Boldface indicates statistical significance at a two-sided p-value of 0.05

All P-values of test for interaction were >0.05

*SD *standard definition, *NC *North Carolina, *CI *confidence interval, *PR *prevalence ratio, *WBGT *Wet Bulb Globe Temperature

**Table 4:** The association between each 1 SD increase in WBGT and sleep health across age groups in NC, PR (95%CI)

	PR (95%CI)					
	Age <60 years (n=575)			Age ≥60 years (n=1,225)		
	2-day WBGT	5-day WBGT	7-day WBGT	2-day WBGT	5-day WBGT	7-day WBGT
Sleep duration						
Absolute	1.01 (0.98-1.03)	1.01 (0.98-1.03)	1.01 (0.98-1.04)	1.00 (0.98-1.02)	1.00 (0.98-1.02)	1.00 (0.98-1.02)
Relative	1.00 (0.98-1.03)	1.01 (0.98-1.03)	1.01 (0.98-1.04)	1.01 (0.99-1.03)	1.01 (0.99-1.03)	1.01 (0.99-1.03)
Daytime sleepiness						
Absolute	1.00 (0.98-1.02)	1.00 (0.98-1.02)	1.00 (0.98-1.02)	0.99 (0.98-1.01)	1.00 (0.98-1.01)	0.99 (0.98-1.01)
Relative	1.00 (0.98-1.02)	1.00 (0.98-1.02)	1.00 (0.98-1.02)	0.99 (0.98-1.01)	1.00 (0.98-1.01)	1.00 (0.98-1.01)
Daytime napping						
Absolute	1.01 (0.99-1.04)	1.02 (0.99-1.04)	1.01 (0.98-1.04)	1.03 (1.01-1.05)	1.02 (1.00-1.04)	1.02 (1.00-1.04)
Relative	1.01 (0.98-1.04)	1.01 (0.98-1.04)	1.01 (0.98-1.03)	1.03 (1.01-1.04)	1.02 (1.00-1.04)	1.01 (0.99-1.03)
Napping duration						
Absolute	1.00 (0.97-1.02)	1.00 (0.98-1.02)	1.00 (0.98-1.02)	1.01 (0.99-1.03)	1.01 (0.99-1.03)	1.01 (0.99-1.02)
Relative	1.00 (0.98-1.02)	1.01 (0.99-1.03)	1.01 (0.99-1.03)	1.00 (0.98-1.02)	1.00 (0.98-1.02)	0.99 (0.98-1.01)

All models were adjusted for age, marital status, educational attainment, and race and ethnicity

Boldface indicates statistical significance at a two-sided p-value of 0.05

All P-values of test for interaction were >0.05

*NC *North Carolina, *CI *confidence interval, *PR *prevalence ratio, *WBGT *Wet Bulb Globe Temperature

### Sensitivity analysis

In the sensitivity analysis, the exposure–response curves between WBGT and sleep were relatively smooth and showed little fluctuation, with no clear J-shaped or U-shaped patterns, suggesting no strong evidence of non-linear associations (Figure S3-S8). Inclusion of female farmers did not materially change the main findings (Tables S3–S4). When redefining short sleep as < 8 h (Table S5-S6), the association between moderate risk WBGT exposure, measured by the 2-day average, and sleep duration < 8 h in Iowa was consistent with the main findings (PR = 1.06 [95% CI: 1.01–1.10], see Table S6). As shown in table S7, altering relative WBGT thresholds also yielded similar results (e.g., 2-day relative WBGT ≥ 90th : PR = 1.02 [1.01–1.04]; ≥95th : PR = 1.02 [1.00–1.03] with daytime napping in NC, which were consistent with the main results using the initial cut point (i.e., 92.5th percentile). Adjusting for daytime napping in models of sleep duration (and vice versa) did not change the findings (Table S8). We did not find a significant association between mean WBGT and a higher prevalence of sleep health problems in either state. Point estimates changed only slightly (e.g., in Iowa, the PR for sleep duration shifted from 1.01 [95% CI: 1.00–1.01] for 2-day absolute WBGT to 1.00 [95% CI: 0.99–1.00] for warm-season average WBGT), supporting the interpretation that the associations between WBGT and sleep outcomes primarily reflect short-term exposure (Table S9).

## Discussion

In this study of farmers in Iowa and NC, heat stress was associated with poor sleep, suggesting a small potential relationship with nighttime sleep duration, daytime napping, and nap duration. Heat stress was linked to a slightly higher likelihood of daytime napping in NC, whereas WBGT levels exceeding the moderate threshold in Iowa were associated with a higher prevalence of short nighttime sleep duration. High heat stress was modestly associated with shorter naps in both states. No effect modification by age was observed. Although there was a weak association between heat stress and sleep, heat stress could affect many agricultural workers as extreme heat events increase in frequency and intensity, potentially signaling elevated sleepiness and increased risks of injury and cardiovascular events.

Our study extends previous research by focusing on farmers who represent a population more exposed than the general population and by using WBGT as a key indicator to assess heat stress. Our results were also comparable to prior literature. For the Iowa sample, moderate risk of heat stress (i.e., 2-day average exposure) was associated with short sleep duration of < 7 h. Our findings were similar with a study in rural Burkina Faso, which observed a positive relationship between nighttime WBGT ≥ 25 °C and shorter sleep duration among 143 participants [13]. But a study including 83 participants from Kenya did not observe a significant correlation between nightly minimum WBGT and sleep duration [14]. These differences might be partially explained by the timing and types of the measurement indicators used (e.g., daytime vs. nighttime; average vs. minimum WBGT). The stronger association with sleep duration seen in Iowa may be due to a higher proportion of participants in Iowa being exposed to lower levels of heat stress and, therefore, may have had lower adaptation to heat upon exposure.

There are several potential explanations for our findings. Heat exposure contributes to increased wakefulness and shorter sleep duration, and humidity further increases wakefulness [35]. Heat adaptation also contributes to the alterations in sleep. Prior studies highlighted that heat-related sleep disruptions persist after 5 days of continuous daytime and nocturnal heat exposure [36]. In our study, the association between moderate risk of heat stress and sleep duration were stronger in Iowa than in NC, despite NC having higher WBGT than Iowa, indicating the need for more attention in regions with less heat adaption. Our sensitivity analyses using a stricter sleep duration cutoff (< 8 h) continued to show an adverse association between heat stress and sleep duration in Iowa, with stronger effect sizes than in the main findings. This suggests that the negative impact of heat stress on sleep duration likely persists even after accounting for potential misreporting or overestimation.

Although prior studies have reported the impact of heat on nightly sleep [6, 8, 20], few have specifically investigated its influence on daytime napping. We observed that daytime napping might would increase with heat exposure, especially in NC. Through heat acclimatization, the body adjusts to high temperatures by sweating and increasing blood flow to the skin [37], yet this process forces the body to expend extra energy. For example, blood vessels dilate to release heat, resulting in lower blood pressure, which can further contribute to fatigue [38]. Napping may serve as a compensatory strategy to mitigate this fatigue [39]. Thus, outdoor workers may feel fatigued but use napping as a strategy to avoid the heat of the day, recover energy, and cool down after finishing their work. This may also explain the lack of association between WBGT and daytime sleepiness in our study. Additionally, high risk to heat stress exposure was associated with shorter napping duration, as seen in NC and Iowa. Heat stress exposure may lead farmers to take naps, but napping duration does not necessarily increase. Consistent with our study, results from a study in Hungary showed that higher daily mean temperatures were associated with reduced total sleep time (the sum of night and napping durations) [7]. Another study suggested that the association between heat and sleep stages are concentrated in the initial segment of the sleep cycle, making it more difficult to fall asleep [40]. Also, while napping may be a strategy to combat fatigue, increased heat stress can hinder heat dissipation, adversely reducing core body cooling and prolonging wakefulness [41]. These combined observations suggest that heat stress can affect napping duration, leading to shorter and possibly poorer quality naps. However, our data are limited in determining whether napping is a direct result of insufficient sleep duration, which warrants further investigation. Nonetheless, our findings extend previous research by reporting an association between heat stress and daytime napping, contributing to a deeper understanding of how heat stress may affect daytime sleep patterns among outdoor workers.

No age-related differences in heat–sleep associations were observed. One possible explanation is that some older participants—such as those over age 70 years -did not meet the analysis inclusion and exclusion criteria, as presented in Table S1. The remaining participants may have had a greater ability to adapt to heat stress compared to those more vulnerable to its effects. Additionally, the sample sizes for each age group in NC were relatively small. This finding requires further investigation.

There are several limitations to consider. First, the cross-sectional design limits causal inference and does not allow for the estimation of lag-specific or cumulative effects that are commonly examined in environmental research. Second, the relatively coarse spatial resolution of the temperature dataset used in this study may have introduced exposure measurement error and attenuated estimates of extreme heat exposure. This limitation was driven by data availability. Future research using higher-resolution temperature data is warranted. Address-based WBGT linkage may misclassify exposure if participants moved, though most farmers are residentially stable. Third, self-reported sleep was not time-referenced, possibly misaligning with heat exposure windows. However, results were consistent across the 3 assessed exposure periods. Fourth, sleep was assessed using self-reported questionnaire items rather than standardized measurement tools; thus, subjective sleep data may be affected by recall bias. We used daytime sleepiness as a proxy for sleep quality, which was imperfect, due to it would be influenced by both sleep quantity and quality. Fifth, since sleep can be influenced by both daytime and nighttime temperatures, future studies should incorporate measurements of both daytime and nighttime temperatures/heat stress. Sixth, some relevant behavioral data were not available such as using working time, air conditioners and drinking ice water which could confound the associations. Future studies including more detailed information on farming work conditions will help distinguish how different agricultural activities are affected by outdoor and indoor environmental heat risks.

Despite the limitations, our study has several strengths. We examined both daytime and nighttime sleep outcomes among outdoor workers—an underrepresented group in sleep research. The use of WBGT offers a more accurate measure of heat stress than ambient temperature. Multiple exposure windows (2-, 5-, and 7-day averages) allowed us to assess acute and short-term effects. Including two states with different climate profiles provides valuable comparative insight into regional differences in heat adaptation and sleep health. The robustness of our findings further supports the association between heat stress and multiple dimensions of sleep health among farmers.

## Conclusion

Short-term heat stress exposure was slightly associated with a higher likelihood of shorter sleep duration among farmers in Iowa and daytime napping among farmers in NC. Additionally, high risk of heat stress was weakly associated with shorter napping duration among farmers in both states. However, the cross-sectional design of the current study does not allow for causal inference. Future studies should use longitudinal data and cohorts of farmers across the US as well as objective sleep indicators to confirm the heat stress and sleep relationship we observed among farmers.

## Supplementary Material

Supplementary Material 1.

## Data Availability

Data are available through approved requests based on Agricultural Health Study policy.The datasets and code used and/or analyzed during the current study are available from the corresponding author on reasonable request.

## Abbreviations

NC: North Carolina
WBGT: Wet bulb globe temperature
PRs: Prevalence ratios
CIs: Confidence Intervals
U.S: United States
HI: Heat Index
AHS: Agricultural Health Study
NARR: North American Regional Reanalysis
NCEP: National Centers for Environmental Prediction
NOAA: National Oceanic and Atmospheric Administration
DAG: Directed acyclic graph
SDs: Standard deviation
