# Supplementary material for: Heat stress in relation to sleep health among farmers: a cross-sectional study
Source: BMC Public Health. 2026 Feb 13;26:933. doi: 10.1186/s12889-026-26614-y (PMC13005482; doi:10.1186/s12889-026-26614-y)
Supplement: Supplementary file 1 — Supplementary Material 1. [file 12889_2026_26614_MOESM1_ESM.docx]

**Supplemental materials**

Excluded

- Did not reside on a farm and perform farm work in the past 12 months (N=10,669)
- Participants were interviewed in other months than May to September (N=4,040)
- Missing key covariate information and sleep data (N=526)
- Missing WBGT data (N=46)

**Total excluded (n =15,281)**

Total AHS Sample in Phase 4, 2013 to 2015 **(N=42,331)**

Final analytic sample adults

**N=8,203**

- 6,403 from Iowa
- 1,800 from NC

Total AHS Sample in Phase 1, 1993 to 1997

**(N=89,655)**

Excluded

- Lost follow-up **(N=47,324)**

Excluded

- Spouse **(N=18,186)**
- Female farmers **(N=661)**

Total AHS male farmers in Phase 4, 2013 to 2015 **(N=23,484)**

**Figure S1. The flow chart of sample selection**

**Table S1. Basic characteristics among excluded and included participants**

|  | **Included:  n=8,203** | **Excluded:  n=15,281** |
| --- | --- | --- |
| **Sociodemographic characteristics** |  |  |
| Age, mean (SD) ^a^ | 63.0 (10.1) | 68.3 (12.1) |
| Missing | 0 | 0 |
| Age categories |  |  |
| <50 | 651 (7.9) | 911 (6.0) |
| 51-60 | 2612 (31.8) | 3019 (19.8) |
| 61-70 | 2762 (33.7) | 4119 (27.0) |
| >70 | 2178 (26.6) | 7232 (47.3) |
| Missing | 0 | 0 |
| Race and ethnicity ^b, c^ |  |  |
| Non-Hispanic White | 8132 (99.1) | 14636 (95.8) |
| Other | 71 (0.9) | 355 (2.3) |
| Missing | 0 | 290 (1.9) |
| Educational attainment ^c^ |  |  |
| 1-8 years | 147 (1.8) | 643 (4.2) |
| Some high school | 198 (2.4) | 761 (5.0) |
| High school graduate or higher | 7858 (95.8) | 13059 (85.5) |
| Missing | 0 | 818 (5.4) |
| Marital status ^d^ |  |  |
| Married/Cohabitating | 7174 (87.5) | 10803 (70.7) |
| Other | 1029 (12.5) | 2007 (13.1) |
| Missing | 0 | 2471 (16.2) |
| Geographical region |  |  |
| Iowa | 6403 (78.1) | 9461 (61.9) |
| NC | 1800 (21.9) | 5668 (37.1) |
| Missing | 0 | 152 (1.0) |
| **Sleep outcomes** |  |  |
| Sleep duration ^e^ |  |  |
| ≥7 hours | 5104 (62.2) | 7906 (51.7) |
| <7 hours | 3099 (37.8) | 4794 (31.4) |
| Missing | 0 | 2581 (16.9) |
| Daytime sleepiness ^f^ |  |  |
| <3 days/week | 7539 (91.9) | 11191 (73.2) |
| ≥3 days/week | 664 (8.1) | 1399 (9.2) |
| Missing | 0 | 2691 (17.6) |
| Daytime napping ^g^ |  |  |
| No | 4543 (55.4) | 6572 (43.0) |
| Yes | 3660 (44.6) | 6130 (40.1) |
| Missing | 0 | 2579 (16.9) |
| Napping duration ^h^ |  |  |
| ≥30 minutes | 1401 (17.1) | 3145 (20.6) |
| <30 minutes | 6802 (82.9) | 9399 (61.5) |
| Missing | 0 | 2737 (17.9) |

Data are presented as count (percentage) or mean (standard deviation)

NC= North Carolina

^a^ Age was measured at Phase 4.

^b^ Other racial and ethnic groups include participants who identified as Non-Hispanic Black, American Indian or Alaska Native, Asian or Pacific Islander, Hispanic/Latino of any race, multiracial, or as 'other' race and measured at Phase 1.

^c^ Educational attainment, race, and ethnicity were reported at Phase 1.

^d^ Other marital status categories included single, divorced or separated, and widowed (measured at Phase 4).

^e^ Sleep duration was assessed by asking participants, “How many hours of sleep do you get each night?”. Participants could respond with: ‘less than 6 hours’, ‘6 hours to 6 hours and 59 minutes’, ’7 hours to 7 hours and 59 minutes’, ‘8 hours to 8 hours and 59 minutes’, and ‘9 hours or more’.

^f^ Daytime sleepiness was assessed by asking participants: “How often do you feel sleepy most of the day?”. Participants could respond with: ‘never’, ‘less than one day per month’,

‘1 to 3 days per month’, 1 to 2 days per week’, ‘3 to 5 days per week’, ‘6 to 7 days per week’.

^g^ Daytime napping was assessed by asking participants, “Do you nap during the day?”. Participants could respond with: ‘yes’, and ‘no’.

^h^  Napping duration was assessed by asking participants, “How long do you nap?”. Participants who reported taking naps were also asked to indicate their napping duration, response was presented as ‘less than 30 minutes’, and ‘more than 30 minutes’.


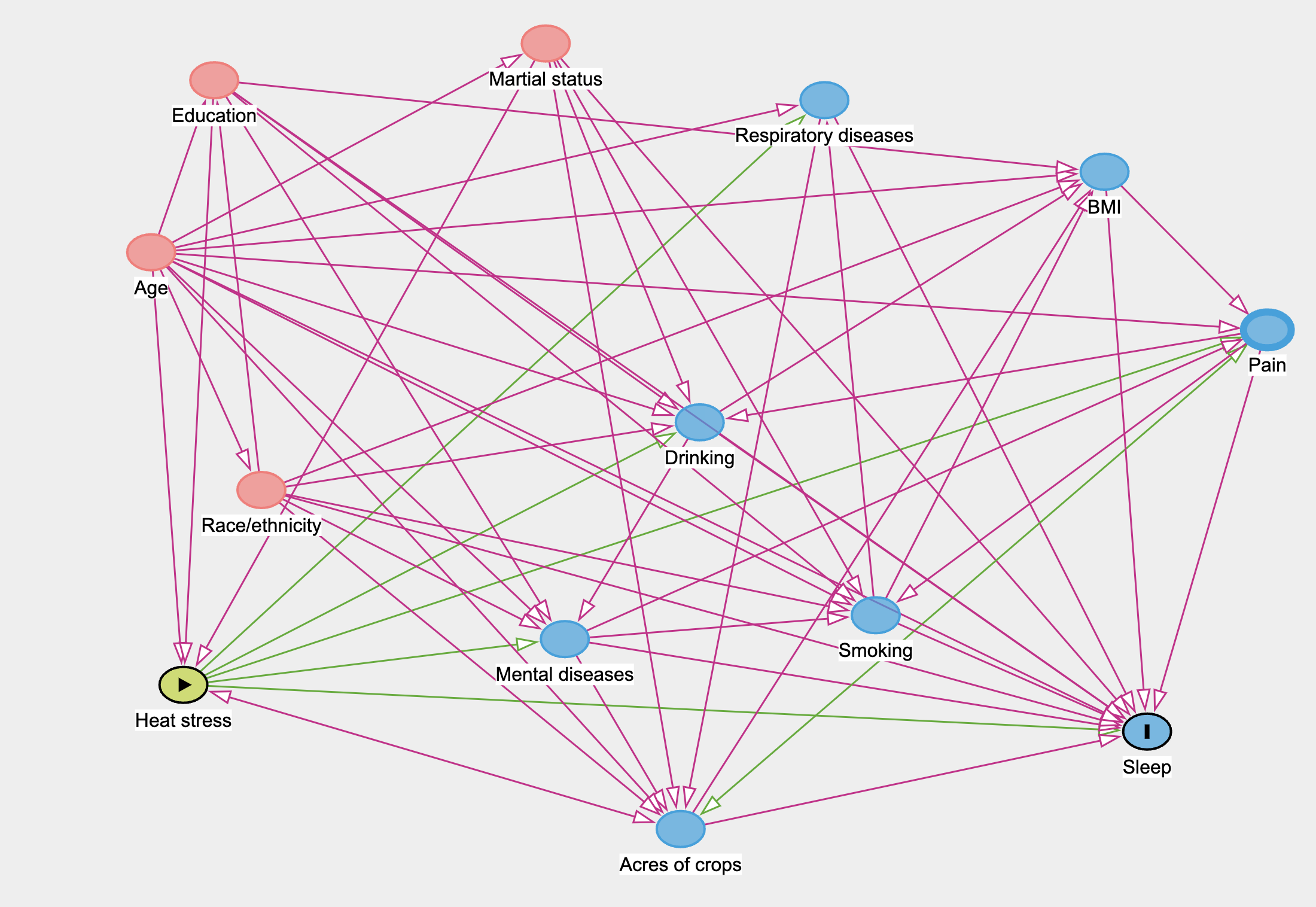


**Figure S2. Directed Acyclic Graph (DAG) for the association between heat stress and sleep**

**Table S2. The association between each 1 SD increase in WBGT and sleep health among male farmers, PR (95%CI).**

|  | PR (95%CI) | | | | | | |
| --- | --- | --- | --- | --- | --- | --- | --- |
|  | Iowa (n=6,403) | | |  | NC (n=1,800) | | |
|  | 2-day WBGT | 5-day WBGT | 7-day WBGT |  | 2-day WBGT | 5-day WBGT | 7-day WBGT |
| Sleep duration | |  |  |  |  |  |  |
| Absolute | 1.01 (1.00-1.01) | 1.00 (1.00-1.01) | 1.01 (1.00-1.01) |  | 1.00 (0.99-1.02) | 1.01 (0.99-1.02) | 1.01 (0.99-1.02) |
| Relative | 1.01 (1.00-1.01) | 1.01 (1.00-1.01) | 1.01 (1.00-1.02) |  | 1.01 (0.99-1.02) | 1.01 (0.99-1.02) | 1.01 (0.99-1.02) |
| Daytime sleepiness | |  |  |  |  |  |  |
| Absolute | 1.00 (0.99-1.00) | 1.00 (0.99-1.00) | 1.00 (0.99-1.00) |  | 0.99 (0.98-1.01) | 1.00 (0.98-1.01) | 1.00 (0.98-1.01) |
| Relative | 0.99 (0.99-1.00) | 0.99 (0.99-1.00) | 1.00 (0.99-1.00) |  | 1.00 (0.98-1.01) | 1.00 (0.99-1.01) | 1.00 (0.98-1.01) |
| Daytime napping | |  |  |  |  |  |  |
| Absolute | 1.01 (1.00-1.01) | 1.01 (1.00-1.02) | 1.01 (1.00-1.01) |  | **1.02 (1.01-1.04)** | **1.02 (1.01-1.04)** | **1.02 (1.00-1.03)** |
| Relative | 1.00 (1.00-1.01) | 1.01 (1.00-1.01) | 1.00 (1.00-1.01) |  | **1.02 (1.00-1.04)** | 1.01 (1.00-1.03) | 1.01 (1.00-1.03) |
| Napping duration | |  |  |  |  |  |  |
| Absolute | 0.99 (0.99-1.00) | 1.00 (0.99-1.00) | 1.00 (0.99-1.01) |  | 1.00 (0.99-1.02) | 1.00 (0.99-1.02) | 1.00 (0.99-1.02) |
| Relative | 0.99 (0.98-1.00) | 1.00 (0.99-1.00) | 1.00 (0.99-1.00) |  | 1.00 (0.99-1.02) | 1.00 (0.99-1.01) | 1.00 (0.99-1.01) |

SD: standard definition; NC: North Carolina; CI: confidence interval; PR: prevalence ratio; WBGT: Wet Bulb Globe Temperature

All models were adjusted for age, marital status, educational attainment, and race and ethnicity.

Boldface indicates statistical significance at a two-sided p-value of 0.05.

**
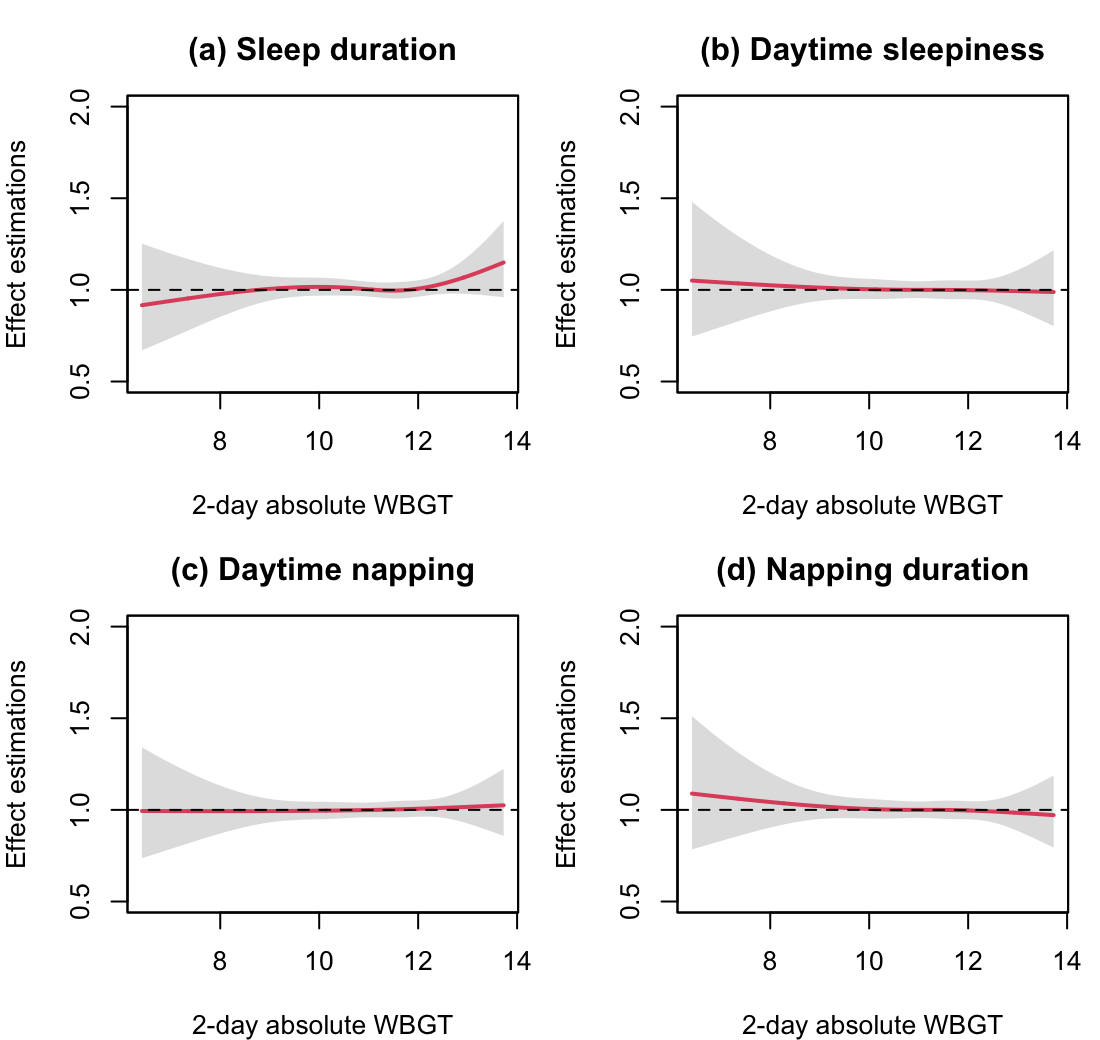

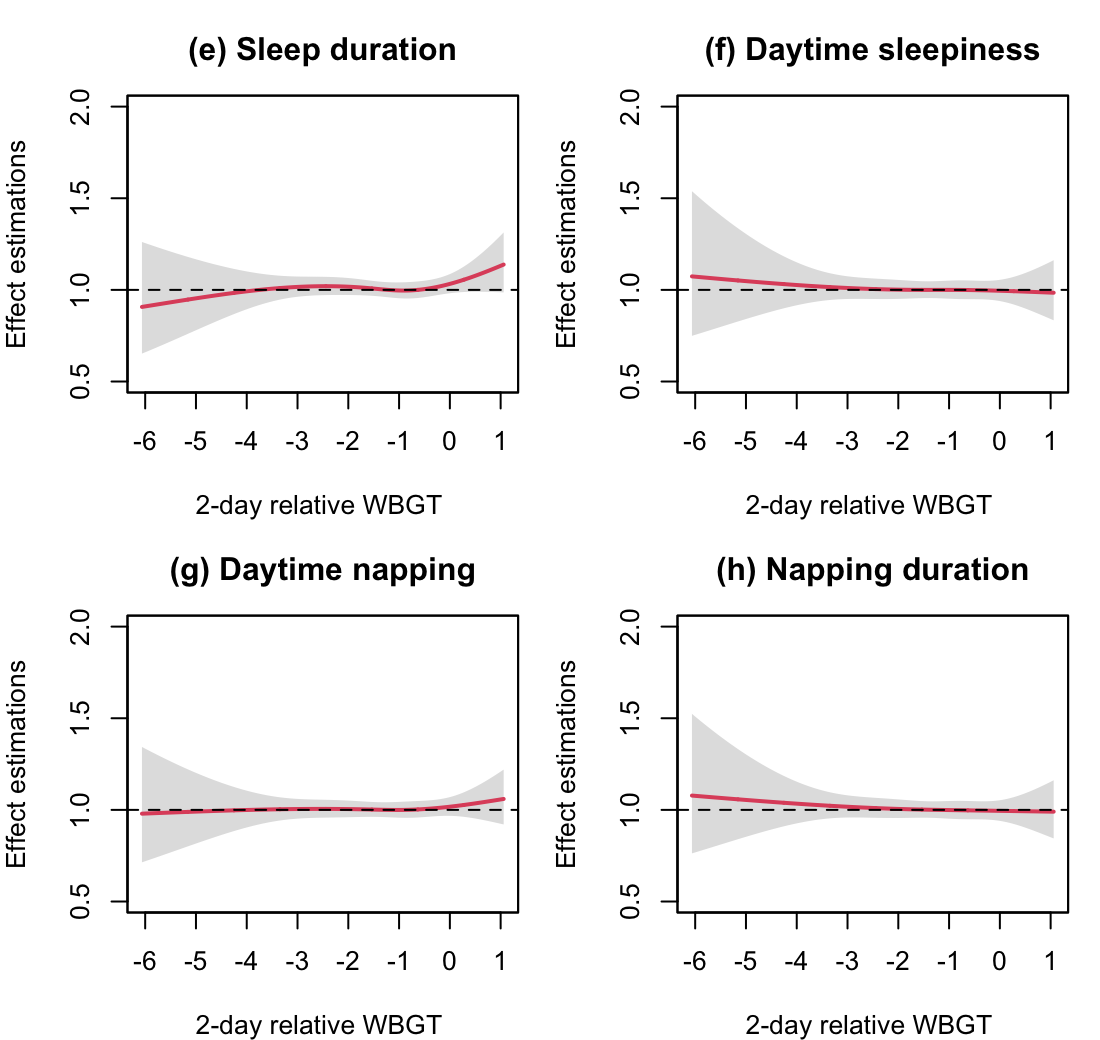
**

**Figure S3. Dose–response associations between 2-day WBGT values and sleep in Iowa**

**
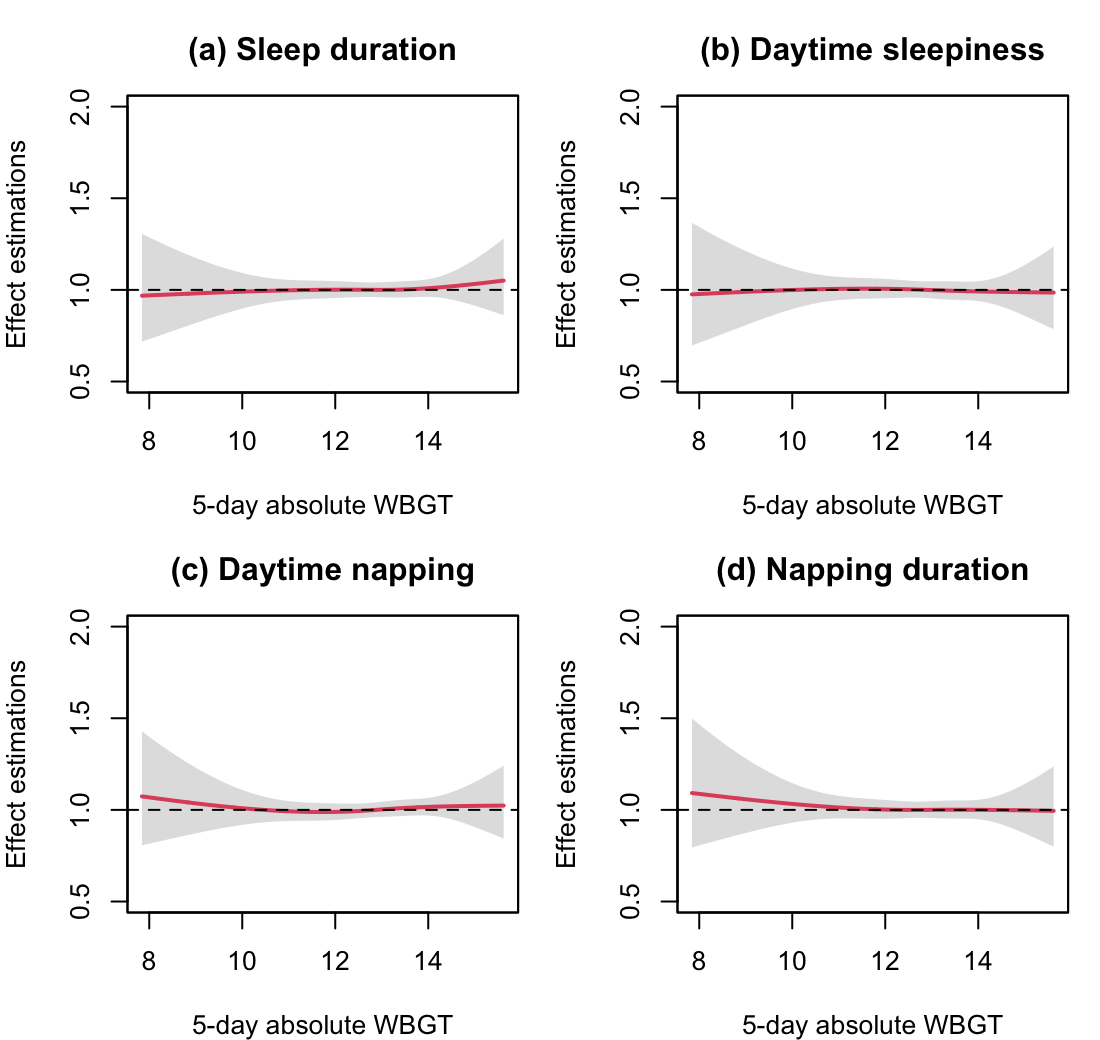

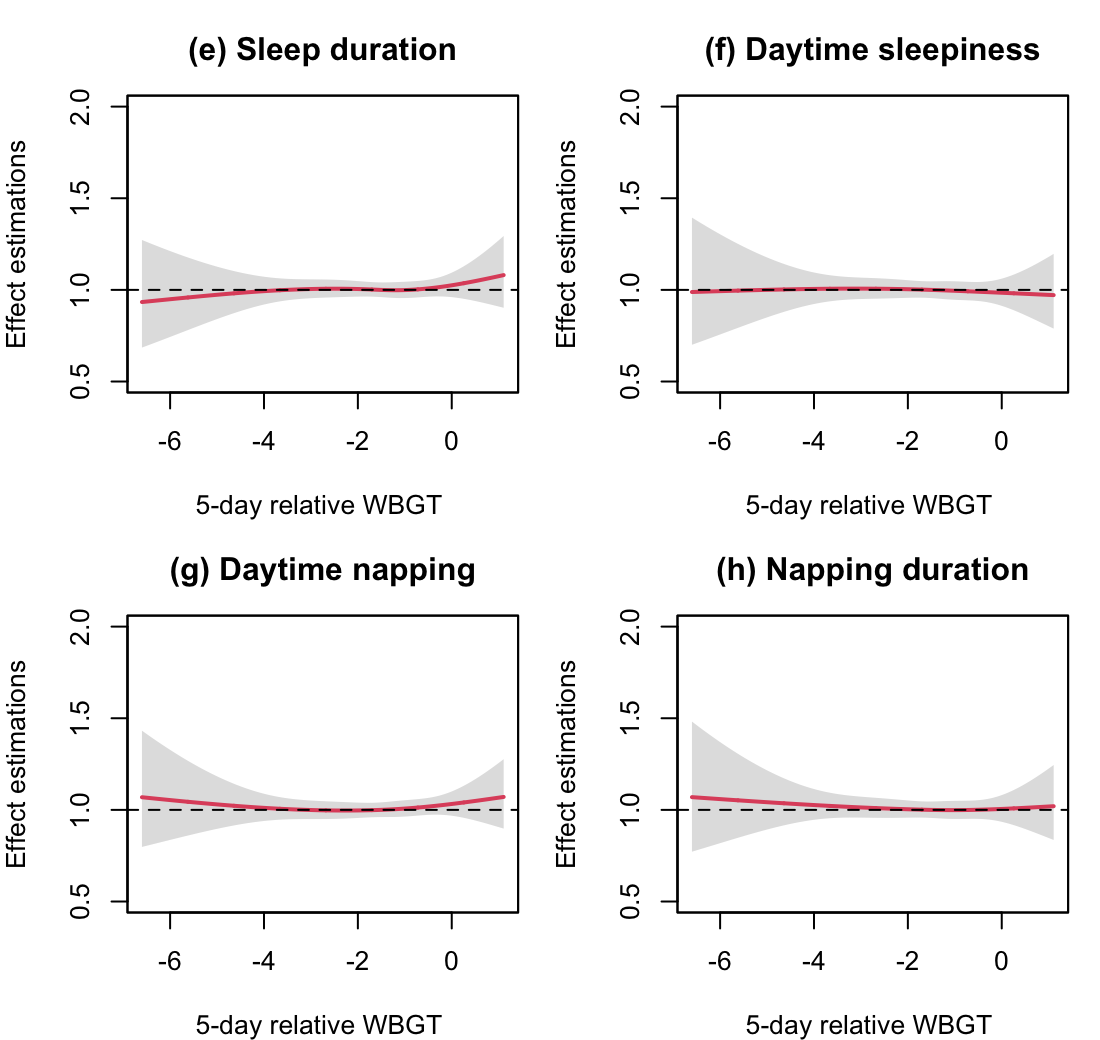
**

**Figure S4. Dose–response associations between 5-day WBGT values and sleep in Iowa**

**
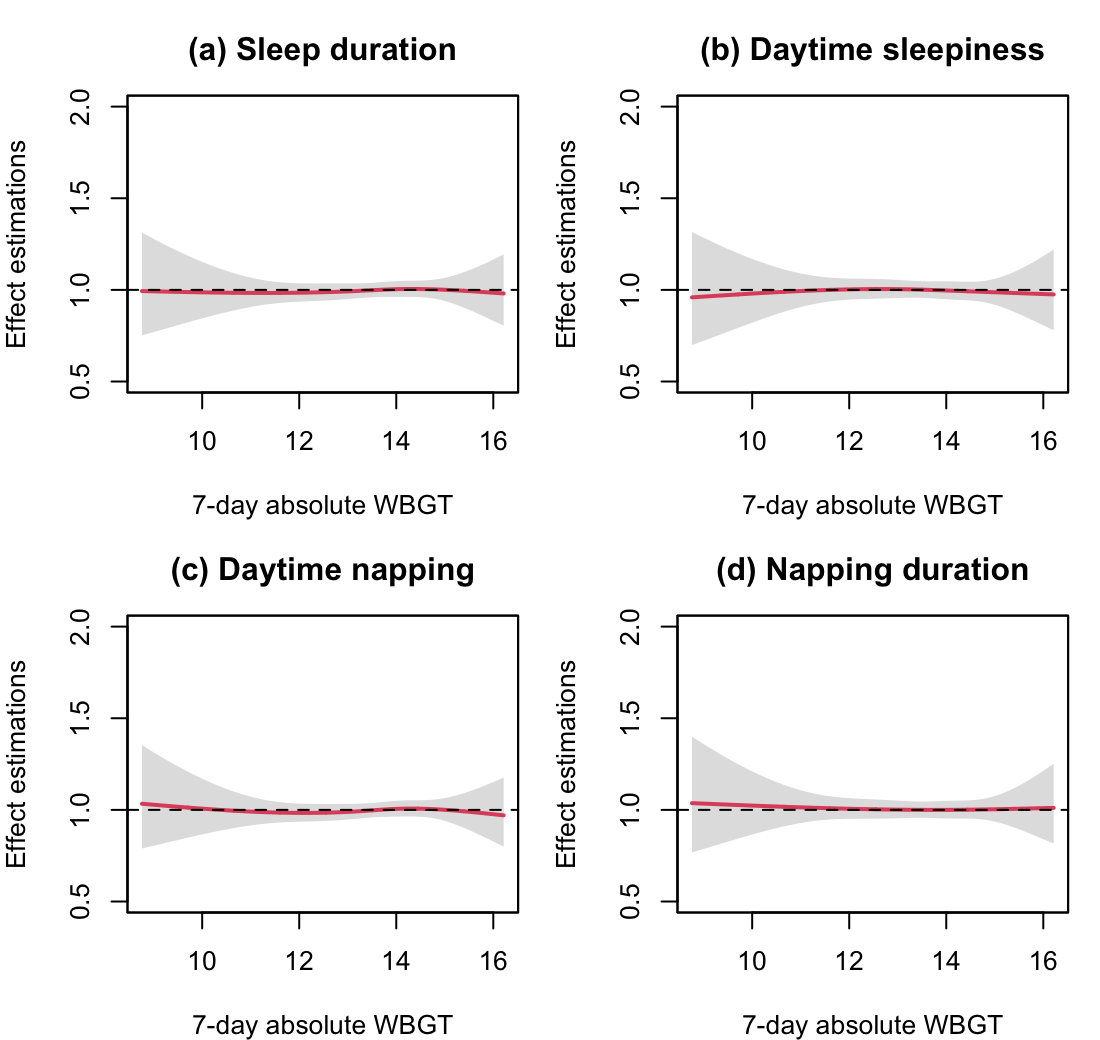

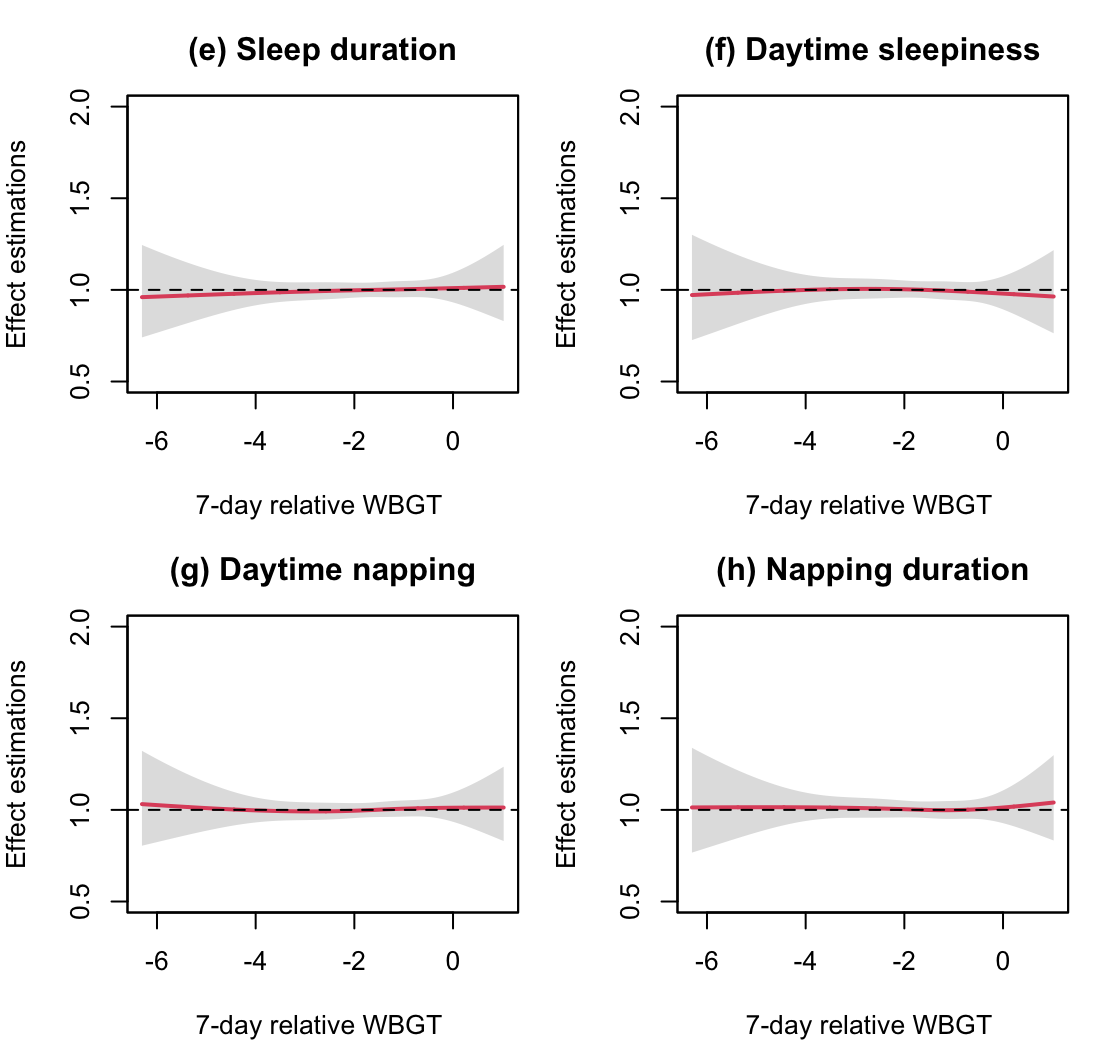
**

**Figure S5. Dose–response associations between 7-day WBGT values and sleep in Iowa**

**
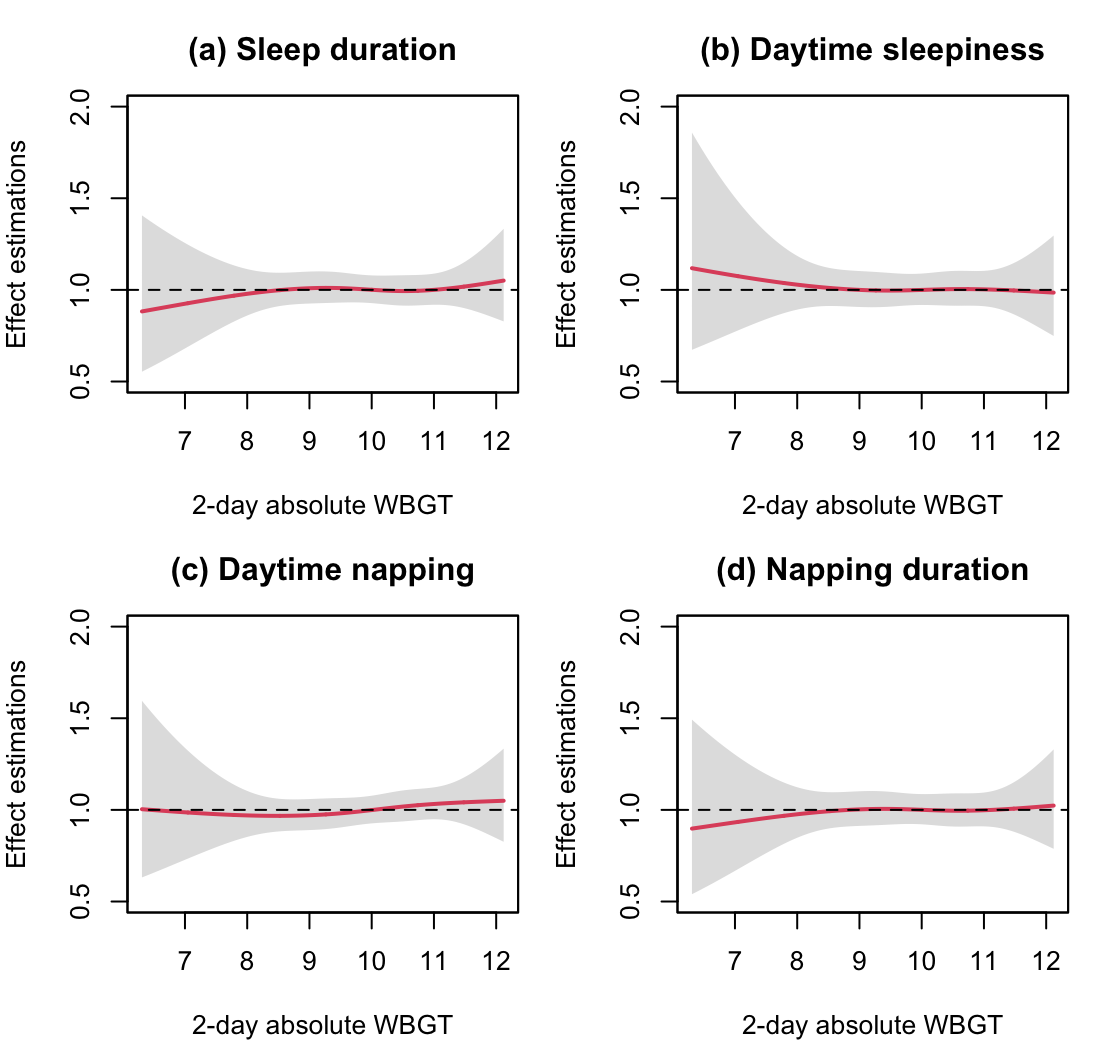

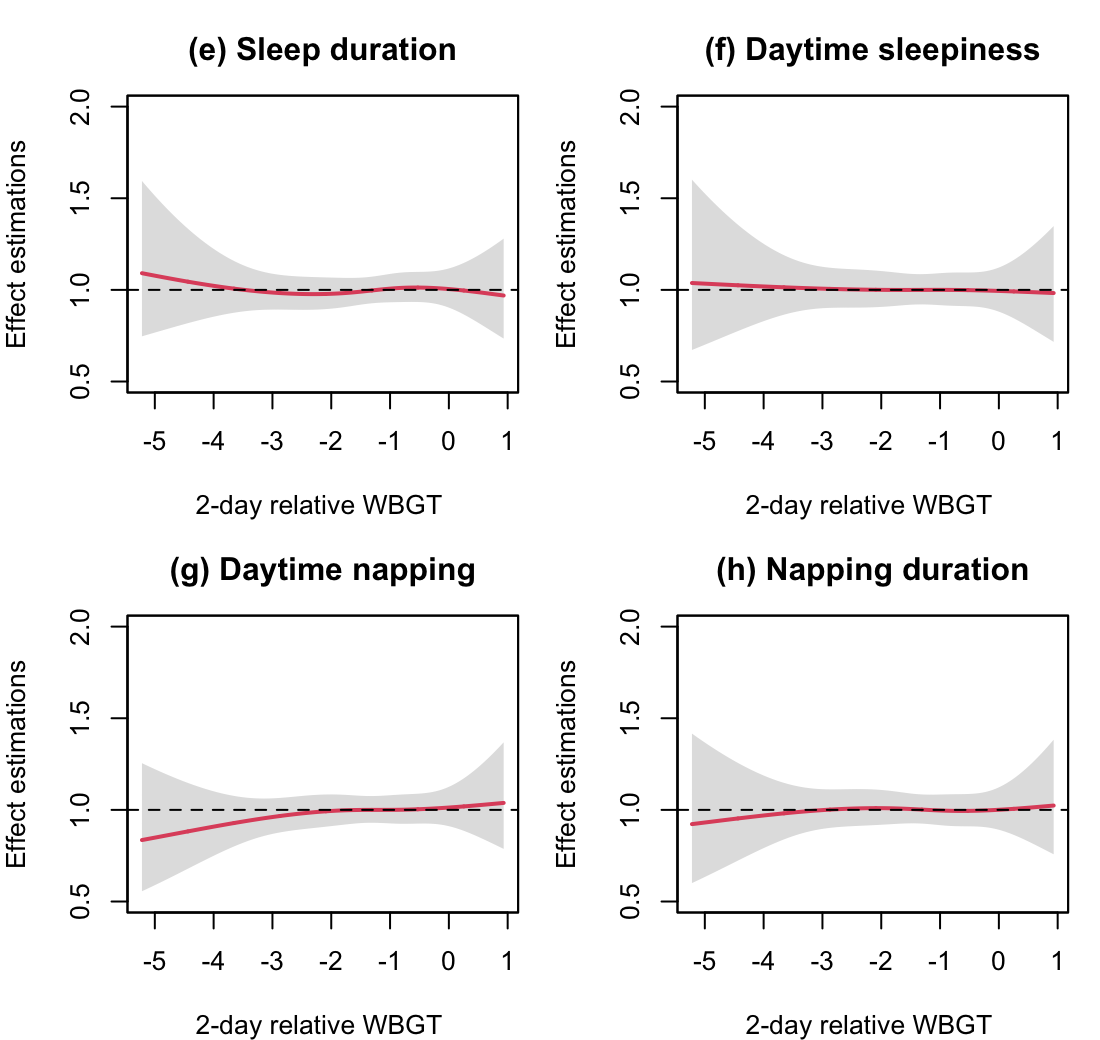
**

**Figure S6. Dose–response associations between 2-day WBGT values and sleep in North Carolina**

**
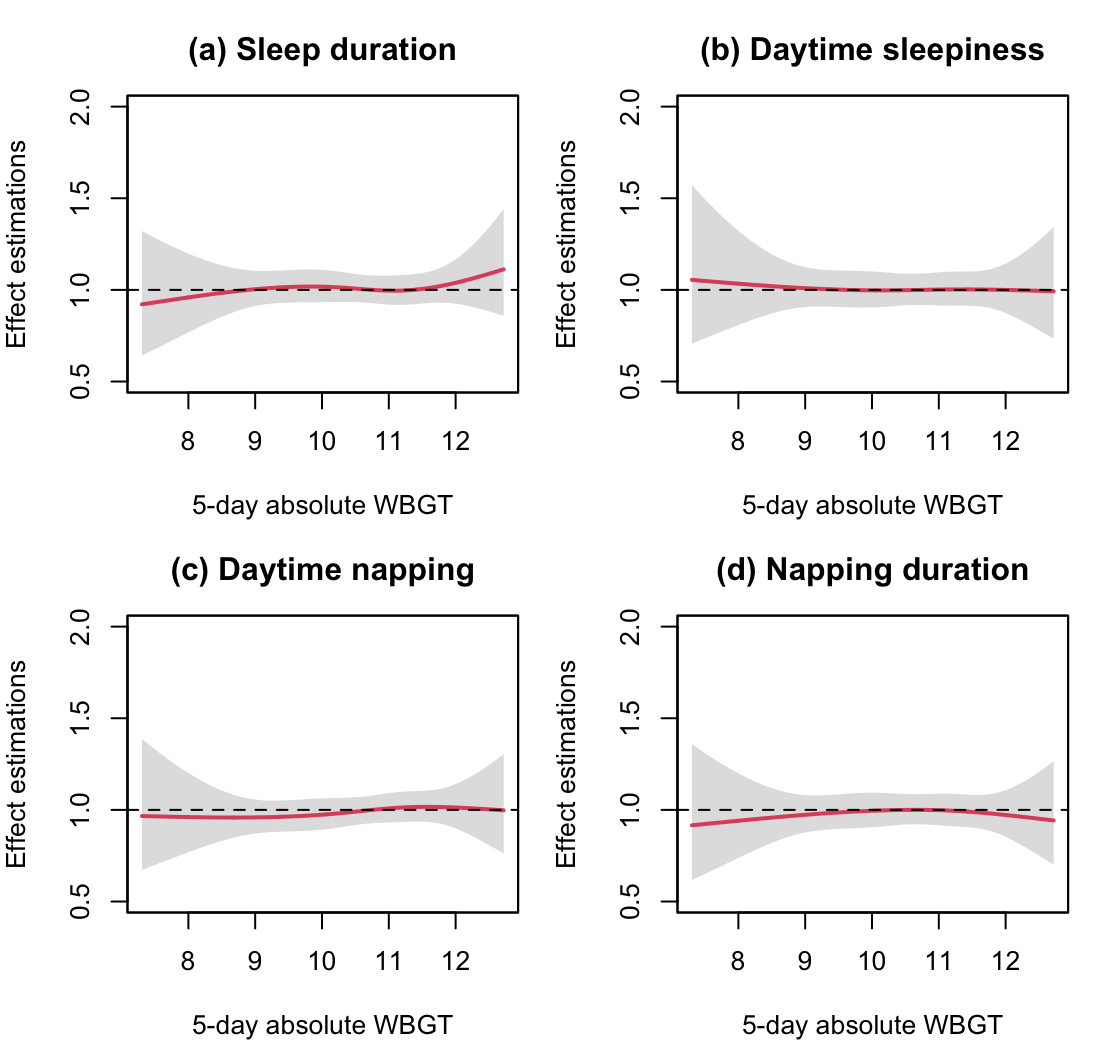

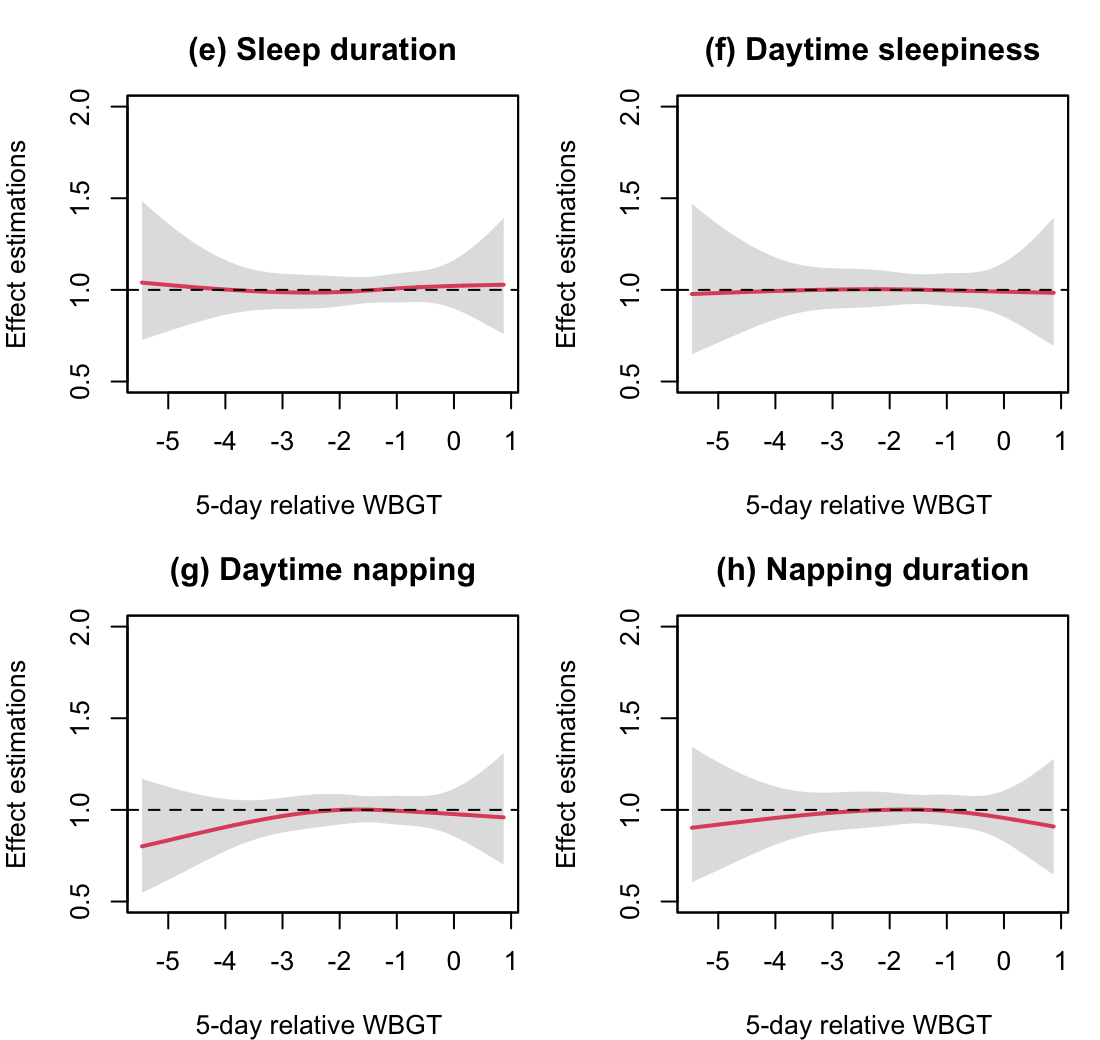
**

**Figure S7. Dose–response associations between 5-day WBGT values and sleep in North Carolina**

**
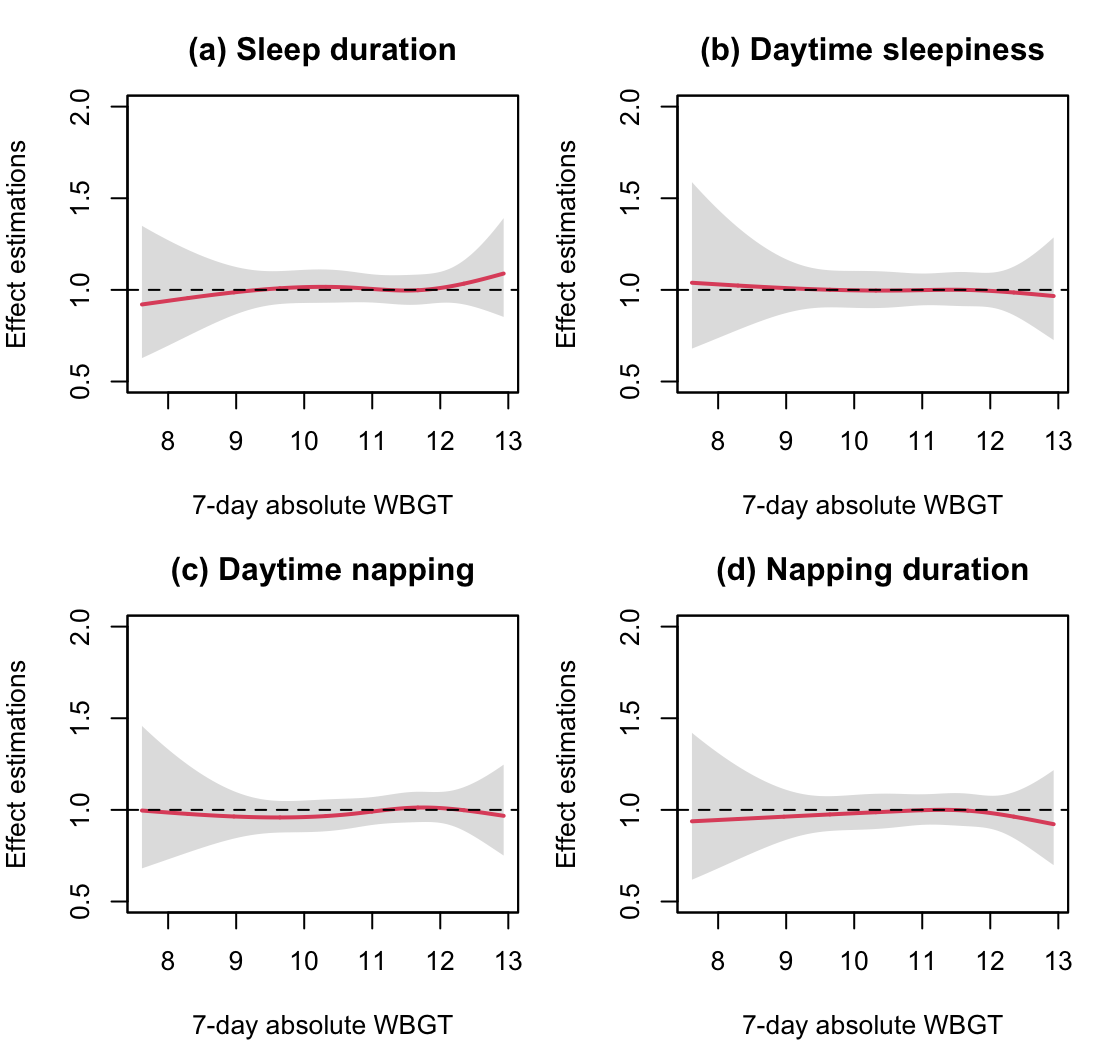

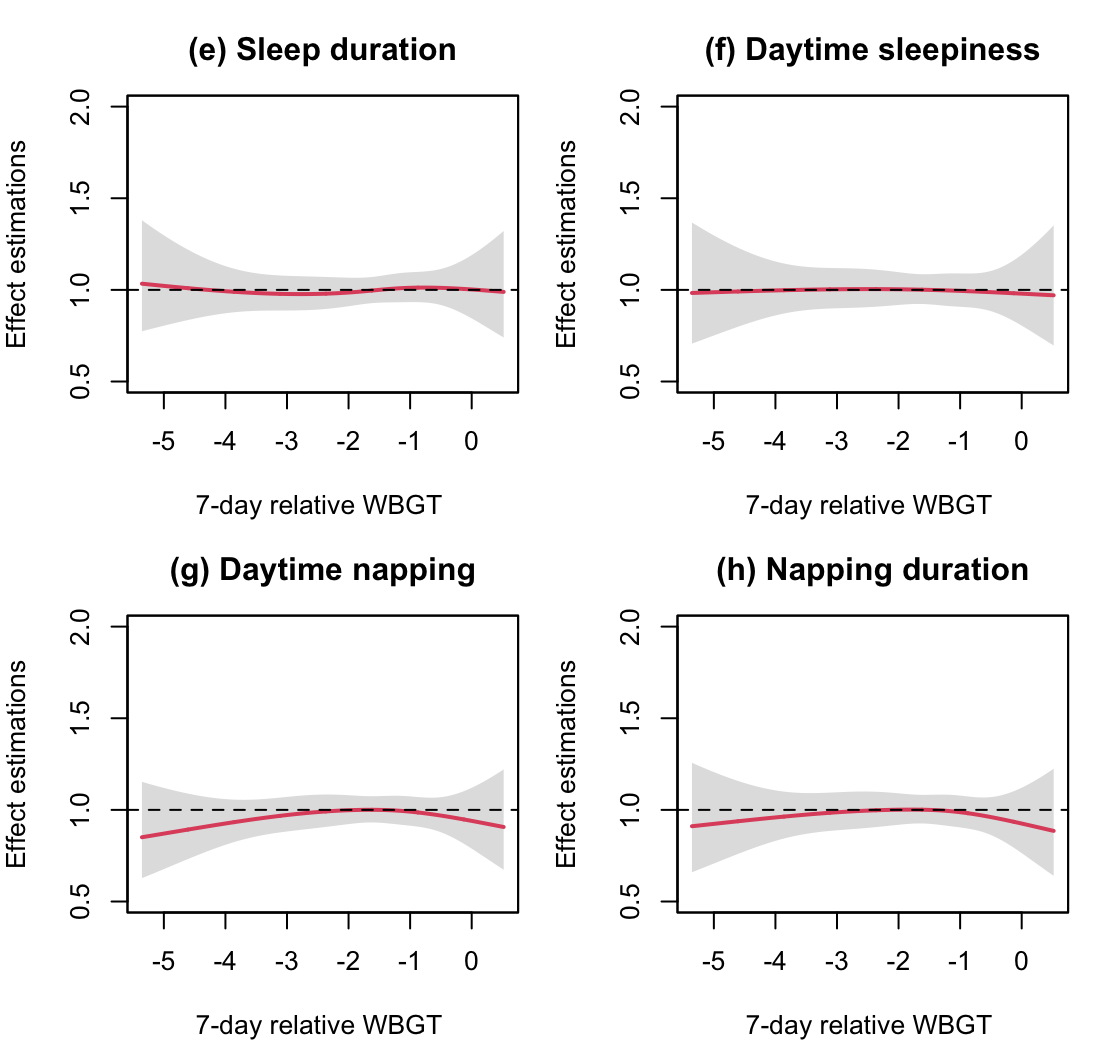
**

**Figure S8. Dose–response associations between 7-day WBGT values and sleep in North Carolina**

**Table S3. The association between each 1 SD increase in WBGT and sleep health among male and female farmers, PR (95%CI).**

|  | PR (95%CI) | | | | | | |
| --- | --- | --- | --- | --- | --- | --- | --- |
|  | Iowa (n=6,499) | | |  | NC (n=1,854) | | |
|  | 2-day WBGT | 5-day WBGT | 7-day WBGT |  | 2-day WBGT | 5-day WBGT | 7-day WBGT |
| Sleep duration | |  |  |  |  |  |  |
| Absolute | 1.00 (1.00-1.01) | 1.00 (0.99-1.01) | 1.01 (1.00-1.01) |  | 1.00 (0.99-1.02) | 1.00 (0.99-1.02) | 1.00 (0.99-1.02) |
| Relative | 1.00 (1.00-1.01) | 1.00 (1.00-1.01) | 1.01 (1.00-1.01) |  | 1.00 (0.99-1.02) | 1.01 (0.99-1.02) | 1.01 (0.99-1.02) |
| Daytime sleepiness | |  |  |  |  |  |  |
| Absolute | 1.00 (0.99-1.00) | 1.00 (0.99-1.00) | 1.00 (0.99-1.00) |  | 1.00 (0.98-1.01) | 1.00 (0.99-1.01) | 1.00 (0.98-1.01) |
| Relative | 0.99 (0.99-1.00) | 0.99 (0.99-1.00) | 1.00 (0.99-1.00) |  | 1.00 (0.98-1.01) | 1.00 (0.99-1.01) | 1.00 (0.99-1.01) |
| Daytime napping | |  |  |  |  |  |  |
| Absolute | 1.00 (1.00-1.01) | 1.01 (1.00-1.02) | 1.01 (1.00-1.01) |  | **1.02 (1.01-1.04)** | **1.02 (1.01-1.04)** | **1.02 (1.00-1.03)** |
| Relative | 1.00 (1.00-1.01) | 1.01 (1.00-1.01) | 1.00 (1.00-1.01) |  | **1.02 (1.00-1.03)** | 1.02 (1.00-1.03) | 1.01 (1.00-1.03) |
| Napping duration | |  |  |  |  |  |  |
| Absolute | 0.99 (0.99-1.00) | 1.00 (0.99-1.00) | 1.00 (0.99-1.01) |  | 1.00 (0.99-1.02) | 1.00 (0.99-1.02) | 1.00 (0.99-1.02) |
| Relative | 0.99 (0.99-1.00) | 1.00 (0.99-1.00) | 1.00 (0.99-1.01) |  | 1.00 (0.99-1.02) | 1.00 (0.99-1.01) | 1.00 (0.99-1.01) |

SD: standard deviation; NC: North Carolina; CI: confidence interval; PR: prevalence ratio; WBGT: Wet Bulb Globe Temperature

All models were adjusted for age, gender, marital status, educational attainment, and race and ethnicity.

Boldface indicates statistical significance at a two-sided p-value of 0.05.

**Table S4. The association between categories of WBGT ^a^ and sleep health among male and female farmers, PR (95%CI).**

|  | PR (95%CI) | | | | | | |
| --- | --- | --- | --- | --- | --- | --- | --- |
|  | Iowa (n=6,499) | | |  | NC (n=1,854) | | |
|  | 2-day WBGT | 5-day WBGT | 7-day WBGT |  | 2-day WBGT | 5-day WBGT | 7-day WBGT |
| Sleep duration |  |  |  |  |  |  |  |
| Moderate risk | **1.03 (1.00-1.07)** | 1.03 (0.98-1.08) | 1.01 (0.94-1.08) |  | 1.00 (0.96-1.04) | 1.00 (0.96-1.03) | 1.02 (0.98-1.05) |
| High risk | 1.05 (0.91-1.22) | 1.04 (0.87-1.24) | 0.91 (0.65-1.27) |  | 1.00 (0.95-1.05) | 1.04 (0.99-1.10) | 1.03 (0.97-1.10) |
| Daytime sleepiness |  |  |  |  |  |  |  |
| Moderate risk | 1.01 (0.98-1.03) | 0.99 (0.96-1.02) | 0.98 (0.94-1.02) |  | 0.99 (0.96-1.02) | 0.99 (0.97-1.02) | 0.99 (0.96-1.02) |
| High risk | 0.97 (0.89-1.06) | 1.00 (0.88-1.13) | 1.16 (0.82-1.64) |  | 0.99 (0.96-1.03) | 1.02 (0.97-1.06) | 0.99 (0.94-1.03) |
| Daytime napping |  |  |  |  |  |  |  |
| Moderate risk | 1.01 (0.98-1.04) | 1.02 (0.97-1.07) | 1.00 (0.94-1.07) |  | **1.04 (1.00-1.08)** | 1.03 (0.99-1.07) | **1.04 (1.00-1.07)** |
| High risk | 0.95 (0.83-1.10) | 1.03 (0.86-1.23) | 1.26 (0.95-1.66) |  | **1.07 (1.02-1.11)** | 1.02 (0.97-1.07) | 1.02 (0.96-1.08) |
| Napping duration |  |  |  |  |  |  |  |
| Moderate risk | 1.00 (0.97-1.03) | 1.00 (0.96-1.05) | 1.02 (0.96-1.08) |  | 1.01 (0.97-1.05) | 1.01 (0.98-1.05) | 1.02 (0.98-1.05) |
| High risk | **0.86 (0.83-0.89)** | **0.89 (0.85-0.92)** | **0.89 (0.86-0.92)** |  | 1.02 (0.98-1.07) | **0.95 (0.91-1.00)** | 0.96 (0.91-1.01) |

NC: North Carolina; CI: confidence interval; PR: prevalence ratio; WBGT: Wet Bulb Globe Temperature

All models were adjusted for age, gender, marital status, educational attainment, and race and ethnicity.

Boldface indicates statistical significance at a two-sided p-value of 0.05.

^a^ Low risk indicates that normal activity is recommended: WBGT <78.8 °F for Iowa and <82.1 °F for NC; moderate risk indicates that planning intense or prolonged activity with discretion is recommended: WBGT 78.8-83.7 °F for Iowa and 82.1-86.0 °F for NC, and high risk indicates that limited or cancelling outdoor activity is recommended: WBGT >83.7 °F for Iowa and >86.0 °F for NC.

**Table S5. The association between each 1 SD increase in WBGT and sleep health among farmers after changing the cut points for sleep duration (<8 hours vs. ≥ 8 hours) and daytime napping (≥1 hour vs. <1 hour), PR (95%CI).**

|  | PR (95%CI) | | | | | | |
| --- | --- | --- | --- | --- | --- | --- | --- |
|  | Iowa (n=6,403) | | |  | NC (n=1,800) | | |
|  | 2-day WBGT | 5-day WBGT | 7-day WBGT |  | 2-day WBGT | 5-day WBGT | 7-day WBGT |
| Sleep duration |  |  |  |  |  |  |  |
| Absolute | 1.01 (1.00-1.02) | 1.01 (1.00-1.02) | 1.01 (1.00-1.02) |  | 0.99 (0.96-1.01) | 0.99 (0.97-1.01) | 0.99 (0.96-1.01) |
| Relative | 1.01 (0.99-1.02) | 1.01 (0.99-1.02) | 1.01 (1.00-1.02) |  | 0.99 (0.97-1.02) | 0.99 (0.97-1.01) | 0.99 (0.97-1.01) |
| Daytime napping |  |  |  |  |  |  |  |
| Absolute | 0.90 (0.76-1.08) | 0.91 (0.75-1.10) | 0.93 (0.77-1.13) |  | 0.88 (0.64-1.21) | 0.95 (0.70-1.29) | 0.94 (0.69-1.27) |
| Relative | 0.88 (0.74-1.04) | 0.88 (0.73-1.05) | 0.90 (0.75-1.08) |  | 0.91 (0.67-1.26) | 1.01 (0.74-1.37) | 0.99 (0.72-1.37) |

SD: standard deviation; NC: North Carolina; CI: confidence interval; PR: prevalence ratio; WBGT: Wet Bulb Globe Temperature

All models were adjusted for age, marital status, educational attainment, and race and ethnicity.

Boldface indicates statistical significance at a two-sided p-value of 0.05.

**Table S6. The association between categories of WBGT ^a^ and sleep health among farmers after changing the cut points for sleep duration (<8 hours vs. ≥ 8 hours) and daytime napping (≥1 hour vs. <1 hour), PR (95%CI).**

|  | | PR (95%CI) | | | | | | |
| --- | --- | --- | --- | --- | --- | --- | --- | --- |
|  | Iowa (n=6,403) | | |  | NC (n=1,800) | | |  |
|  | 2-day WBGT | 5-day WBGT | 7-day WBGT |  | 2-day WBGT | 5-day WBGT | 7-day WBGT |  |
| Sleep duration |  |  |  |  |  |  |  |  |
| Moderate risk | **1.06 (1.01-1.10)** | 1.04 (0.98-1.11) | 1.04 (0.96-1.13) |  | 0.99 (0.93-1.05) | 0.98 (0.93-1.04) | 1.00 (0.95-1.06) |  |
| High risk | 0.90 (0.70-1.18) | 0.88 (0.64-1.21) | 0.92 (0.53-1.59) |  | 0.97 (0.91-1.05) | 1.00 (0.93-1.08) | 0.98 (0.89-1.07) |  |
| Daytime napping |  |  |  |  |  |  |  |  |
| Moderate risk | 1.01 (0.51-2.01) | 0.97 (0.31-3.04) | 1.15 (0.29-4.62) |  | 0.72 (0.31-1.69) | 1.07 (0.52-2.20) | 1.06 (0.53-2.12) |  |
| High risk | -* | -* | -* |  | 0.90 (0.34-2.38) | 0.51 (0.12-2.15) | 0.67 (0.16-2.80) |  |

SD: standard deviation; NC: North Carolina; CI: confidence interval; PR: prevalence ratio; WBGT: Wet Bulb Globe Temperature

^a^ Low risk indicates that normal activity is recommended: WBGT <78.8 °F for Iowa and <82.1 °F for NC; moderate risk indicates that planning intense or prolonged activity with discretion is recommended: WBGT 78.8-83.7 °F for Iowa and 82.1-86.0 °F for NC, and high risk indicates that limited or cancelling outdoor activity is recommended: WBGT >83.7 °F for Iowa and >86.0 °F for NC.

All models were adjusted for age, marital status, educational attainment, and race and ethnicity.

Boldface indicates statistical significance at a two-sided p-value of 0.05.

* Not estimable due to small sample size.

**Table S7. The association between each 1 SD increase in relative heat stress and sleep health among farmers after changing the thresholds for relative WBGT, PR (95%CI).**

|  | PR (95%CI) | | | | | | |
| --- | --- | --- | --- | --- | --- | --- | --- |
|  | Iowa (n=6,403) | | |  | NC (n=1,800) | | |
|  | 2-day WBGT | 5-day WBGT | 7-day WBGT |  | 2-day WBGT | 5-day WBGT | 7-day WBGT |
| Sleep duration | |  |  |  |  |  |  |
| 90th percentile | 1.01 (1.00-1.01) | 1.01 (1.00-1.01) | 1.01 (1.00-1.02) |  | 1.01 (0.99-1.02) | 1.01 (0.99-1.02) | 1.01 (0.99-1.02) |
| 95th percentile | 1.01 (1.00-1.02) | 1.01 (1.00-1.01) | 1.01 (1.00-1.02) |  | 1.01 (0.99-1.02) | 1.01 (0.99-1.02) | 1.01 (0.99-1.02) |
| Daytime sleepiness | |  |  |  |  |  |  |
| 90th percentile | 0.99 (0.99-1.00) | **0.99 (0.99-1.00)** | 0.99 (0.99-1.00) |  | 1.00 (0.98-1.01) | 1.00 (0.99-1.01) | 1.00 (0.98-1.01) |
| 95th percentile | 0.99 (0.99-1.00) | 0.99 (0.99-1.00) | 1.00 (0.99-1.00) |  | 1.00 (0.98-1.01) | 1.00 (0.99-1.01) | 1.00 (0.98-1.01) |
| Daytime napping | |  |  |  |  |  |  |
| 90th percentile | 1.00 (1.00-1.01) | 1.01 (1.00-1.01) | 1.00 (1.00-1.01) |  | **1.02 (1.01-1.04)** | **1.02 (1.00-1.03)** | 1.01 (1.00-1.03) |
| 95th percentile | 1.00 (1.00-1.01) | 1.01 (1.00-1.01) | 1.00 (1.00-1.01) |  | **1.02 (1.00-1.03)** | 1.01 (1.00-1.03) | 1.01 (0.99-1.02) |
| Napping duration | |  |  |  |  |  |  |
| 90th percentile | **0.99 (0.98-1.00)** | 0.99 (0.99-1.00) | 1.00 (0.99-1.00) |  | 1.00 (0.99-1.02) | 1.00 (0.99-1.02) | 1.00 (0.99-1.01) |
| 95th percentile | **0.99 (0.98-1.00)** | 0.99 (0.99-1.00) | 1.00 (0.99-1.00) |  | 1.00 (0.99-1.02) | 1.00 (0.98-1.01) | 1.00 (0.98-1.01) |

SD: standard deviation; NC: North Carolina; CI: confidence interval; PR: prevalence ratio; WBGT: Wet Bulb Globe Temperature

All models were adjusted for age, marital status, educational attainment, and race and ethnicity.

Boldface indicates statistical significance at a two-sided p-value of 0.05.

**Table S8. The association between each 1 SD increase in WBGT and sleep health among male farmers after adjusting for sleep duration or daytime napping, PR (95%CI).**

|  | PR (95%CI) | | | | | | |
| --- | --- | --- | --- | --- | --- | --- | --- |
|  | Iowa (n=6,403) | | |  | NC (n=1,800) | | |
|  | 2-day WBGT | 5-day WBGT | 7-day WBGT |  | 2-day WBGT | 5-day WBGT | 7-day WBGT |
| Sleep duration ^a^ | |  |  |  |  |  |  |
| Absolute | 1.00 (1.00-1.01) | 1.00 (1.00-1.01) | 1.01 (1.00-1.02) |  | 1.00 (0.99-1.02) | 1.01 (0.99-1.02) | 1.01 (0.99-1.02) |
| Relative | 1.01 (1.00-1.01) | 1.01 (1.00-1.01) | 1.01 (1.00-1.02) |  | 1.00 (0.99-1.02) | 1.01 (0.99-1.02) | 1.01 (0.99-1.02) |
| Daytime napping ^b^ | |  |  |  |  |  |  |
| Absolute | 1.00 (1.00-1.01) | 1.01 (1.00-1.02) | 1.01 (1.00-1.01) |  | **1.02 (1.01-1.04)** | **1.02 (1.01-1.04)** | **1.02 (1.00-1.03)** |
| Relative | 1.00 (1.00-1.01) | 1.01 (1.00-1.01) | 1.00 (1.00-1.01) |  | **1.02 (1.00-1.04)** | 1.01 (1.00-1.03) | 1.01 (1.00-1.03) |

SD: standard deviation; NC: North Carolina; CI: confidence interval; PR: prevalence ratio; WBGT: Wet Bulb Globe Temperature

^a^ Models were adjusted for age, gender, marital status, educational attainment, race and ethnicity, and daytime napping.

^b^ Models were adjusted for age, gender, marital status, educational attainment, race and ethnicity, and sleep duration.

Boldface indicates statistical significance at a two-sided p-value of 0.05.

**Table S9. The association between** **each 1 SD increase in mean warm-season WBGT and sleep health among male farmers, PR (95%CI).**

|  | PR (95%CI) | | |
| --- | --- | --- | --- |
|  | Iowa (n=6,403) ^a^ |  | NC (n=1,800) ^b^ |
| Sleep duration | 1.00 (0.99-1.00) |  | 1.00 (0.99-1.02) |
| Daytime sleepiness | 1.00 (1.00-1.01) |  | 1.00 (0.99-1.01) |
| Daytime napping | 1.01 (1.00-1.02) |  | 1.01 (0.99-1.03) |
| Napping duration | 1.01 (0.99-1.01) |  | 1.01 (0.99-1.02) |

SD: standard deviation; NC: North Carolina; CI: confidence interval; PR: prevalence ratio; WBGT: Wet Bulb Globe Temperature

All models were adjusted for age, gender, marital status, educational attainment, and race and ethnicity.

Boldface indicates statistical significance at a two-sided p-value of 0.05.

^a^ The mean (SD) warm season WBGT was 66.9 (1.51)°F.

^b^ The mean (SD) warm season WBGT was 74.3 (4.99)°F.
